# HESD: A Database of Candidate Reactions and Enzymes for Metabolic Engineering of *Corynebacterium glutamicum*

**DOI:** 10.3390/ijms27219593

**Published:** 2026-10-27

**Authors:** Timofey V. Ivanisenko, Pavel S. Demenkov, Maksim A. Kleshchev, Artur S. Venzel, Tamara M. Khlebodarova, Anastasia R. Volyanskaya, Nikolay A. Kolchanov, Vladimir A. Ivanisenko

**Affiliations:** Institute of Cytology and Genetics, Siberian Branch of the Russian Academy of Sciences, Novosibirsk 630090, Russia; itv@bionet.nsc.ru (T.V.I.); demps@bionet.nsc.ru (P.S.D.); max82cll@bionet.nsc.ru (M.A.K.); venzel@bionet.nsc.ru (A.S.V.); tamara@bionet.nsc.ru (T.M.K.); a.volianskaia@g.nsu.ru (A.R.V.);

**Keywords:** database, metabolic engineering, heterologous expression, *Corynebacterium glutamicum*, genome-scale metabolic model, flux balance analysis, Rhea

## Abstract

The effect of a heterologous enzyme depends on the position of its reaction within the host metabolic network. We present HESD, a database of candidate reactions for heterologous introduction into *Corynebacterium glutamicum* ATCC 13032 with assessments of network position and feasibility. Rhea release 141, ChEBI and UniProtKB/Swiss-Prot data were integrated with the genome-scale model iCGB21FR, linking reaction variants to 32,619 protein records, with taxonomic assignments for 32,577 records from 3149 taxa. HESD contains 846 variants with both main substrate–product pair metabolites represented in the model and 3642 with one represented metabolite. Variants were classified topologically and assessed for stoichiometric balance, flux feasibility and energy-generating cycles. After excluding variants forming energy-generating cycles, 406 of 525 fully mapped variants retained feasible flux under loopless flux balance analysis. Topological classification agreed for 87.5% of variants shared with the independent model iCW773. Reaction RHEA:79573, catalyzed by ArcE from *Thermococcus kodakarensis*, replaced two consecutive steps catalyzed by ArgG and ArgH and increased predicted maximum L-arginine flux by 22.7% in iCGB21FR supplemented with Ddh and 12.5% in iCW773 with native Ddh, at a minimum growth rate of 0.05 h^−1^. HESD supports candidate selection for modeling and experimental validation.

## 1. Introduction

Modern industrial production of amino acids and other biologically active compounds relies, to a large extent, on microbial synthesis using producer strains with purposefully modified metabolism. Rational metabolic engineering makes it possible to enhance target product formation, reduce by-product formation and balance the activities of central and specialized metabolic pathways [1]. One of the most widely used organisms for such tasks is *Corynebacterium glutamicum*. Owing to its well-characterized metabolism, the available methods of genetic modification and its capacity to accumulate amino acids, this species is widely used for the production of L-glutamate, L-lysine, other amino acids and valuable low-molecular-weight compounds [1,2]. Further increases in the productivity of *C. glutamicum* strains require the coordinated modification of central and target metabolism, metabolite transport and cellular regulation.

One metabolic engineering strategy is the heterologous expression of genes encoding enzymes that enable additional biochemical reactions to proceed in the cell of the host microorganism [3]. Heterologous expression is most often used to assemble a multi-step pathway towards a preselected compound. At the same time, the introduction of a single heterologous enzyme can also substantially alter the metabolic phenotype: it can enable the formation of a new product, replace several consecutive steps, change energy and cofactor requirements, or redistribute reducing equivalents [4,5,6,7,8].

The functional outcome of such an intervention depends on the chemical equation of the added reaction, the availability of its substrates and cosubstrates, the possibility of further use of its products and competition with native reactions. Depending on these factors, the introduced reaction may remain blocked with respect to flux, may form an alternative route, or may change the flux distribution within the metabolic network [9].

Most computational methods for designing heterologous pathways start from a predefined target product. They search for pathways leading from metabolites of the recipient organism to that product and rank the resulting solutions by length, stoichiometric yield, thermodynamic feasibility or enzyme availability [10,11,12,13]. Such methods are indispensable for specific production tasks; on their own, however, they do not provide a systematic, product-independent comparison of known single reactions against the entire metabolic network of a given organism. As a result, identifying reactions that could create new connections between metabolic pathways, shorten existing sequences of conversions or alter the cofactor balance requires, for every individual task, a separate search in specialized information resources followed by evaluation using metabolic modeling.

Genome-scale metabolic models (GEMs) make it possible to represent the set of known reactions of an organism within a single stoichiometric system. For *C. glutamicum*, the models iCGB21FR and iCW773 have been developed, as well as the enzyme-constrained model ecCGL1 [14,15,16] and the software module fluxMicrobiotech, designed to evaluate the metabolic potential of mutant strains under different cultivation conditions and genetic modifications [17]. Flux balance analysis makes it possible to determine feasible steady-state reaction rate distributions and to estimate the theoretical limits of target product formation [9].

The predictive power of a GEM is determined both by the completeness and correctness of the metabolic reconstruction and by the agreement of the imposed constraints with the physiological state of the cell under study. Kulyashov et al. showed that integrating transcriptomic data and cultivation conditions into a context-specific model changes the predicted flux distribution and helps to refine the metabolic capabilities of the organism under study [18]. A comparative analysis of genome-scale metabolic reconstruction strategies, in turn, revealed differences in the composition and predictive power of models built using different information resources, reconstruction algorithms and gap-filling procedures [19]. These findings justify the need to consider both the quality of the underlying reconstruction and the biological context when modeling the consequences of metabolic interventions.

Matching a biochemical reaction to a metabolic model requires consistent information on the chemical identity of compounds, on stoichiometry and on the catalytic function of proteins. Relationships between genes, enzymes, reactions and metabolic pathways are represented in KEGG [20]; curated biochemical reaction equations are provided by Rhea [21], an ontology of chemical compounds by ChEBI [22], and expert-curated annotations of proteins and the reactions they catalyze by UniProtKB/Swiss-Prot [23]. However, identifiers, protonation states and the level of detail with which compounds are described may differ between these resources and genome-scale models. Cross-references and structural descriptors are used to reconcile such data, as implemented, for example, in MetaNetX/MNXref [24].

A separate problem arises when the metabolic network is represented as a graph. Water, protons, adenine and nicotinamide cofactors and other common participants take part in a large number of reactions; using them as ordinary graph vertices leads to biologically uninformative shortest paths. Representing a reaction as substrate–product pairs formed with regard to the transfer of the molecular backbone makes it possible to separate the principal chemical conversion from the participation of cofactors and other auxiliary compounds [25].

The aim of the present work was to create the Heterologous Enzyme Shortcut Discovery (HESD) database, containing directed reaction candidates selected against the Rhea annotations of the iCGB21FR model of *C. glutamicum* ATCC 13032, with curated nativity decisions and associated enzyme records, in order to evaluate possible bioengineering modifications of host metabolism.

To populate, validate and subsequently update the HESD database, we developed the HESD computational system, which integrates data from Rhea, ChEBI and UniProtKB/Swiss-Prot with the metabolic model of the host organism. Each reaction was analyzed separately in the forward and the reverse direction; for each directed variant we determined the main substrate–product pair, its relationship to the paths of the source model, its full stoichiometry, its flux feasibility and its association with annotated enzymes.

A web interface was developed to access HESD, providing search by reactions, metabolites, enzymes and external identifiers, as well as the combined application of filters by topological class and by the results of the stoichiometric assessment. The interface allows the relationships between database objects and the data sources to be inspected, and selected records to be exported for subsequent modeling and experimental validation. The results of the flux assessment are stored in the database and can be combined with the remaining filters, so that a candidate list can be restricted to reactions shown to carry flux in the host model.

Application of the developed approach yielded the HESD database, comprising 846 directed reaction variants for which both metabolites of the main substrate–product pair are represented in the model and 3642 variants for which only one of them is represented. The variants are linked to 32,619 UniProtKB/Swiss-Prot protein records, 32,577 of which have taxonomic assignments from 3149 taxa. Of the 525 variants with full stoichiometry that did not give rise to internal energy-generating cycles, 406 retained feasible flux under loopless FBA. Modeling of selected examples showed that a single added reaction can make a new product accessible, as for chorismate pyruvate lyase UbiC, or redistribute reducing equivalents between NADH and NADPH, as for GapA and GapN [7,8]. Reaction RHEA:79573, catalyzed by ArcE [26], can replace two consecutive steps of L-arginine synthesis; after restoration of the native Ddh reaction absent from the original reconstruction [27], its addition increased the maximum L-arginine flux by 22.7%. In the independent reconstruction iCW773 [15], the topological class agreed for 87.5% of the shared variants, and the effect of ArcE was reproduced with a smaller magnitude (+12.5%).

## 2. Results

### 2.1. General Characteristics and Stages of HESD Database Construction

**Figure 1:**
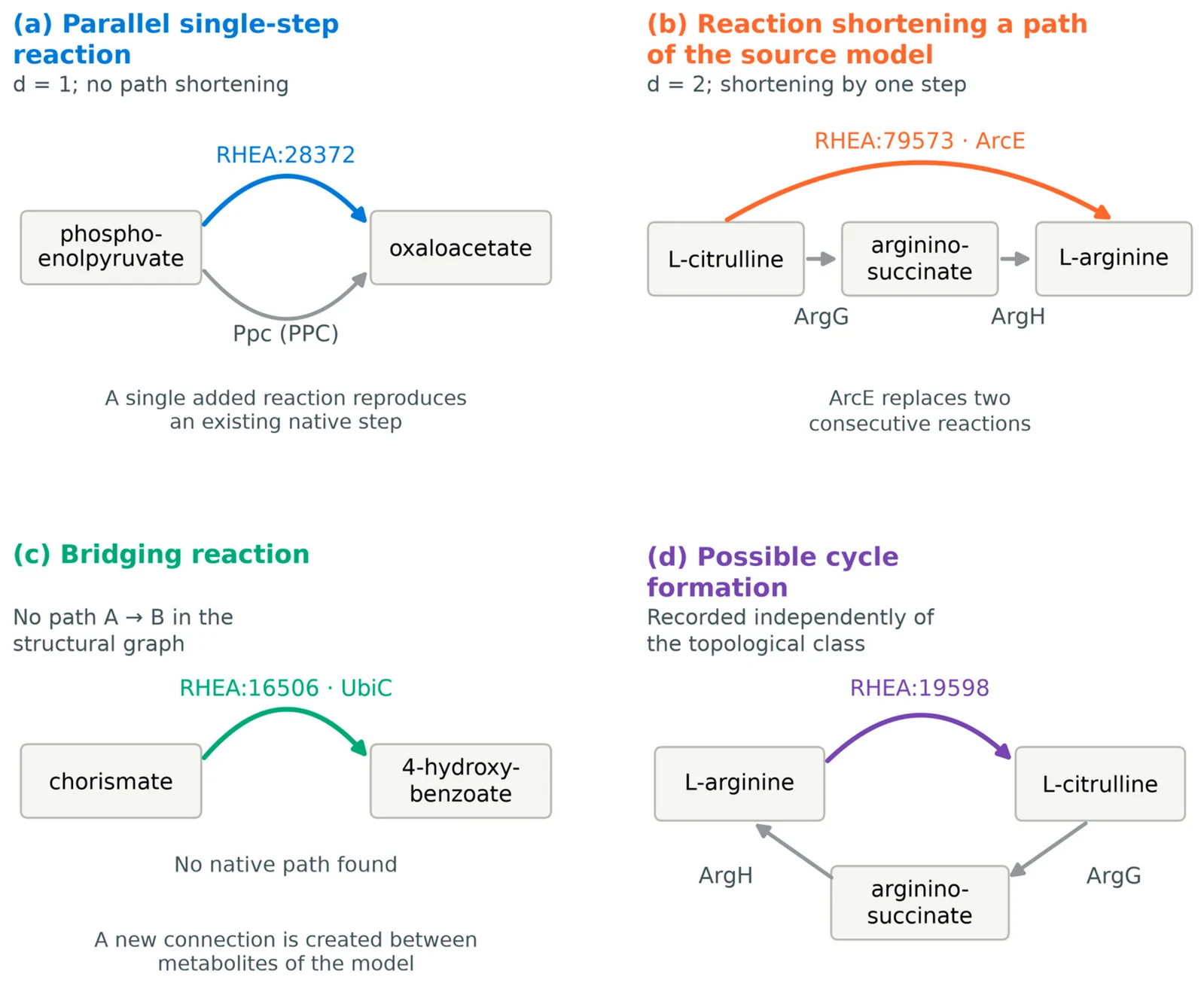
Classification of directed reaction variants by their position relative to the paths of the source model. Gray arrows denote reactions of iCGB21FR, colored arrows denote the added reactions under analysis. Three mutually exclusive classes are shown: a parallel single-step reaction (**a**), a reaction shortening a path of the source model (**b**), and a bridging reaction (**c**). The possibility of cycle formation (**d**) is recorded independently and does not constitute a fourth class. ArgG and ArgH denote argininosuccinate synthase and argininosuccinate lyase; the corresponding reactions in the models are ARGSS and ARGSL.

The HESD database contains records of biochemical reactions that passed an initial filtering step against the Rhea annotations of the iCGB21FR model of *Corynebacterium glutamicum* ATCC 13032 and are regarded as candidates for heterologous introduction. Each HESD record characterizes a particular reaction direction and combines information on the chemical conversion, its position within the metabolic network, the results of the computational assessment and the associated enzymes. Depending on how completely the main substrate–product pair could be matched to the model, the records are divided into reaction variants with two or with one matched metabolite. Reaction variants with two matched metabolites are classified with respect to the native metabolic network represented in iCGB21FR as parallel single-step reactions, as reactions shortening a path in the source model, or as bridging reactions. The classification principle for directed reaction variants is shown schematically in Figure 1 and described below. The size of these groups and the results of the subsequent checks are presented in the following subsections.

The colored arrows in Figure 1 correspond to the added directed reaction variants. In general terms, such a variant was denoted A → B and used as the unit of analysis and as an individual HESD record. For each source Rhea reaction, the forward and the reverse conversion were considered separately; in the variant A → B, compound A acts as the substrate and B as the product. A and B form the main substrate–product pair that characterizes the central chemical conversion within the complete equation. The main pair was identified using an approximation of heavy-atom conservation based on elemental composition; explicit atom mapping was not performed [25,28]. Water, protons, common cofactors and other auxiliary compounds were excluded only from the set of candidates for the role of main metabolites. Among the remaining participants, the main substrate A and the corresponding main product B were selected as the pair with the highest estimated fraction of shared heavy atoms according to elemental composition. Consequently, A and B are participants of the same reaction and do not denote consecutive steps of a multi-step metabolic pathway. The complete equation with all participants was retained and used in the subsequent assessment of stoichiometry and flux feasibility. Two situations were treated separately. A compound on the list of auxiliary participants may itself be the donor or the acceptor of the transferred fragment; such variants were excluded from the exact analysis. A redox couple whose members share an elemental composition, such as Fe^2+^ and Fe^3+^, attains the highest score the measure can return and displaces the conversion of interest; both iron ions were therefore included in the list of auxiliary compounds. Quality control of the main-pair assignment is described in Section 2.4.

The gray arrows in Figure 1 denote the reactions of the directed graph built from the cytosolic reactions of iCGB21FR. The vertices of this structural graph were metabolites, and the directed edges corresponded to the conversions of main pairs as defined by the equations and by the SBML reversibility attribute. For variant A → B, the shortest directed path from A to B was determined, and its length d was defined as the number of reactions it comprises. The sequence of iCGB21FR reactions forming this path is referred to below as the path of the source model. The topological class was determined by the presence and the length of this path. At d = 1, iCGB21FR already contained a single-step conversion between the metabolites of the main pair, and the added variant was therefore assigned to the parallel single-step reactions (Figure 1a). At d > 1, a single added reaction replaced a sequence of two or more model reactions; such a variant was assigned to the reactions shortening a path of the source model (Figure 1b). If no directed path from A to B existed, the variant was classified as a bridging reaction, potentially creating a new connection between regions of the metabolic network (Figure 1c). These three classes are mutually exclusive. The presence in the source model of a path in the opposite direction, from B to A, was recorded independently as a flag indicating the possible formation of a cycle upon addition of the reaction (Figure 1d); this flag does not constitute a separate class.

**Figure 2:**
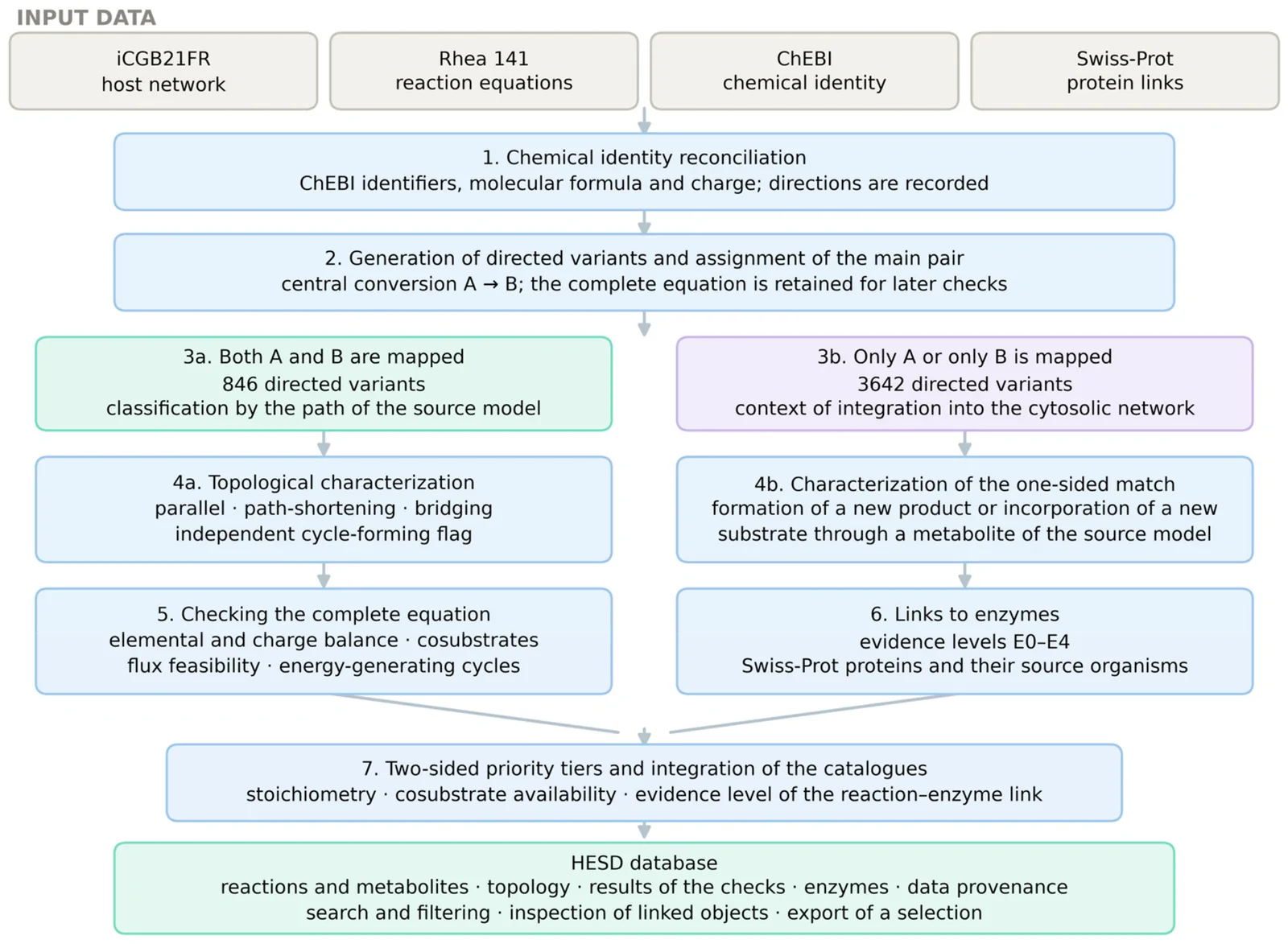
General scheme of HESD database construction. The data sources used and the successive stages are shown: reconciliation of the chemical identity of compounds, extraction of the main substrate–product pair, matching to iCGB21FR metabolites, classification and validation of directed variants, establishment of links to enzymes, assignment of the priority tier and generation of database records.

The HESD database was populated using the HESD computational system, which sequentially matches information on reactions, metabolites and enzymes to the metabolic model of the host organism and stores the results of each stage. The overall sequence of database construction is presented in Figure 2. It includes the preparation of the source data, reconciliation of the chemical identity of compounds, extraction of directed variants and main pairs, matching to the *C. glutamicum* model, determination of the position of the reaction relative to the paths of the model, stoichiometric and flux assessment, establishment of links to enzymes and storage of the results in HESD.

In the first stage, a harmonized set of source data was assembled. The iCGB21FR model defined the composition of the metabolic network of *C. glutamicum* ATCC 13032 against which the added reactions were analyzed. Rhea served as the source of curated stoichiometric equations [21], ChEBI as the source of information on the chemical identity and structure of the compounds participating in the reactions [22], and UniProtKB/Swiss-Prot as the source of curated links between reactions and proteins [23]. The output of this stage was a set of reaction equations, descriptions of their participants and associated proteins, brought to a common representation and provided with provenance information. The versions of the resources and the detailed inclusion criteria are given in the Section 4 and in Table S1.

In the next stage, correspondences were established between the reaction participants from Rhea and the metabolites of iCGB21FR. By chemical identity matching we mean the assignment of records from different sources to the same chemical compound on the basis of ChEBI identifiers and the consistency of chemical descriptors. This stage is necessary because one compound may have different identifiers or be represented in different protonation states. Ambiguous correspondences were not used in the exact analysis. Candidates sharing a Rhea master transformation with the model, or covered by the curated native-equivalence exclusions, were removed. Therefore, the definition of a reaction as potentially “heterologous” refers to the composition of the chosen metabolic model and does not in itself prove the absence of the corresponding enzymatic activity in *C. glutamicum*.

Directed variants were then generated for each reaction and the main substrate–product pair was determined. This representation made it possible to compare the central conversion with the directed paths of the model while retaining the remaining participants for the subsequent analysis of the complete equation. If the main pair could not be unambiguously separated from a cofactor conversion, or if several pairs had equivalent scores, the variant was not included in the exact analysis.

Reactions in which both the main substrate and the main product belong to the list of auxiliary compounds used here—for example, the conversion of NAD^+^ into NADP^+^ by NAD kinase—are not assigned a main pair and are not included in the exact catalogue. Such enzymes may offer opportunities for improving biosynthesis, but their reactions fall outside the scope of the chosen network representation. The replacement of a native enzyme by a more efficient heterologous enzyme catalyzing the same chemical conversion, and the differences in their kinetic properties, are not evaluated in the current version of HESD.

Once the main substrate–product pair had been determined, we established whether A and B were represented among the metabolites of iCGB21FR. Variants for which both metabolites were matched were designated as variants with a two-sided match of the main pair; if a correspondence was found only for A or only for B, they were designated as variants with a one-sided match. These terms characterize the completeness with which the components of the main pair were matched and are unrelated to the reversibility of the reaction. For variants with a two-sided match, the path from A to B in the source model can be determined and the topological class established. Variants with a one-sided match were retained as potential ways of forming a product absent from the model from an available substrate, or of incorporating a new substrate into the metabolic network through a product represented in it.

Topological classification required only the two metabolites of the main pair to be matched to the model, whereas incorporation of the complete equation into iCGB21FR was possible only when all reaction participants were matched. The complete equation was checked for elemental and charge balance and for the presence in the model of the required cosubstrates and cofactors. For reactions that could be transferred into the model with full stoichiometry, we determined whether a non-zero steady-state flux was possible and checked whether the addition of the reaction gave rise to internal energy-generating cycles. Thus, the topological class characterizes the position of the added conversion within the graph but does not guarantee its flux feasibility or a useful metabolic effect.

Links between reactions and enzymes were characterized by evidence levels from E0 to E4, reflecting the transition from experimentally confirmed and curated links to predictions based on chemical structure transformations or structural similarity. Level E4 hypotheses were stored separately from the results of exact matching. The priority tier was assigned taking into account stoichiometric correctness, cosubstrate availability and the level of evidence for the link between reaction and enzyme. It characterizes the completeness and the soundness of the information available for a variant, but not the magnitude of its possible influence on target product formation. Detailed criteria are given in Section 4.

In the final stage, the results were combined into HESD records. For each directed variant, the source equation, the main substrate–product pair, the results of matching to iCGB21FR, the topological characteristics, the results of the stoichiometric check, and information on the associated proteins and on the data sources were stored. Retaining the source and intermediate information ensures that the classification can be verified and allows the database to be rebuilt when the source resources or the host model are updated. Each HESD record for a two-sided directed variant includes the flux assessment results: whether its full stoichiometry could be transferred into the host model, whether the addition gave rise to an internal energy-generating cycle, whether a non-zero flux is possible under ordinary and under loopless FBA, the flux capacity obtained, and whether that value is a proven maximum or a certified lower bound. Variants without a flux assessment are distinguished from variants classified as unable to carry flux. The per-variant calculations are provided in TSV/JSON format and in the Supplementary table workbook, linked to the catalogue by ID.

### 2.2. Construction and Composition of the HESD Database

The iCGB21FR model contains 1042 metabolites and 1539 reactions. Unambiguous correspondences to ChEBI records were established for 719 metabolites. After merging records belonging to the same chemical compound in different cellular compartments, 477 unique compounds were represented in the cytosol of the model. When the directed graph was built, transport, exchange and other reactions not corresponding to intracellular enzymatic conversions were excluded. Main pairs could be determined for 428 model reactions, which involved 366 iCGB21FR metabolites.

The initial selection comprised a subset of Rhea release 141 consisting of 27,994 directed reaction variants linked to curated Swiss-Prot proteins. After applying the model Rhea annotations and curated nativity decisions, and excluding variants with an ambiguously determined main pair, 846 directed reaction variants with two matched metabolites of the main pair were retained in HESD. They corresponded to 423 chemical conversions irrespective of direction. A further 3642 directed variants contained one main metabolite that was represented both in the model and in the graph of cytosolic reactions. For 478 additional reaction variants, one metabolite was matched to the model but its connection to the cytosolic graph could not be established; these variants were not included in the group of one-sided matched records.

The reaction variants included in HESD are associated with 32,619 unique UniProtKB/Swiss-Prot proteins. Taxonomic assignments are available for 32,577 proteins from 3149 sources; the remaining 42 accessions are unresolved in the frozen taxonomy snapshot. For the 846 variants in which both metabolites of the main pair are represented in iCGB21FR, 10,915 associated proteins from 1413 sources were identified, and for the 3642 variants with one represented metabolite, 22,501 proteins from 2918 sources. The proteins and taxonomic sources associated with these two groups of variants partially overlap.

**Figure 3:**
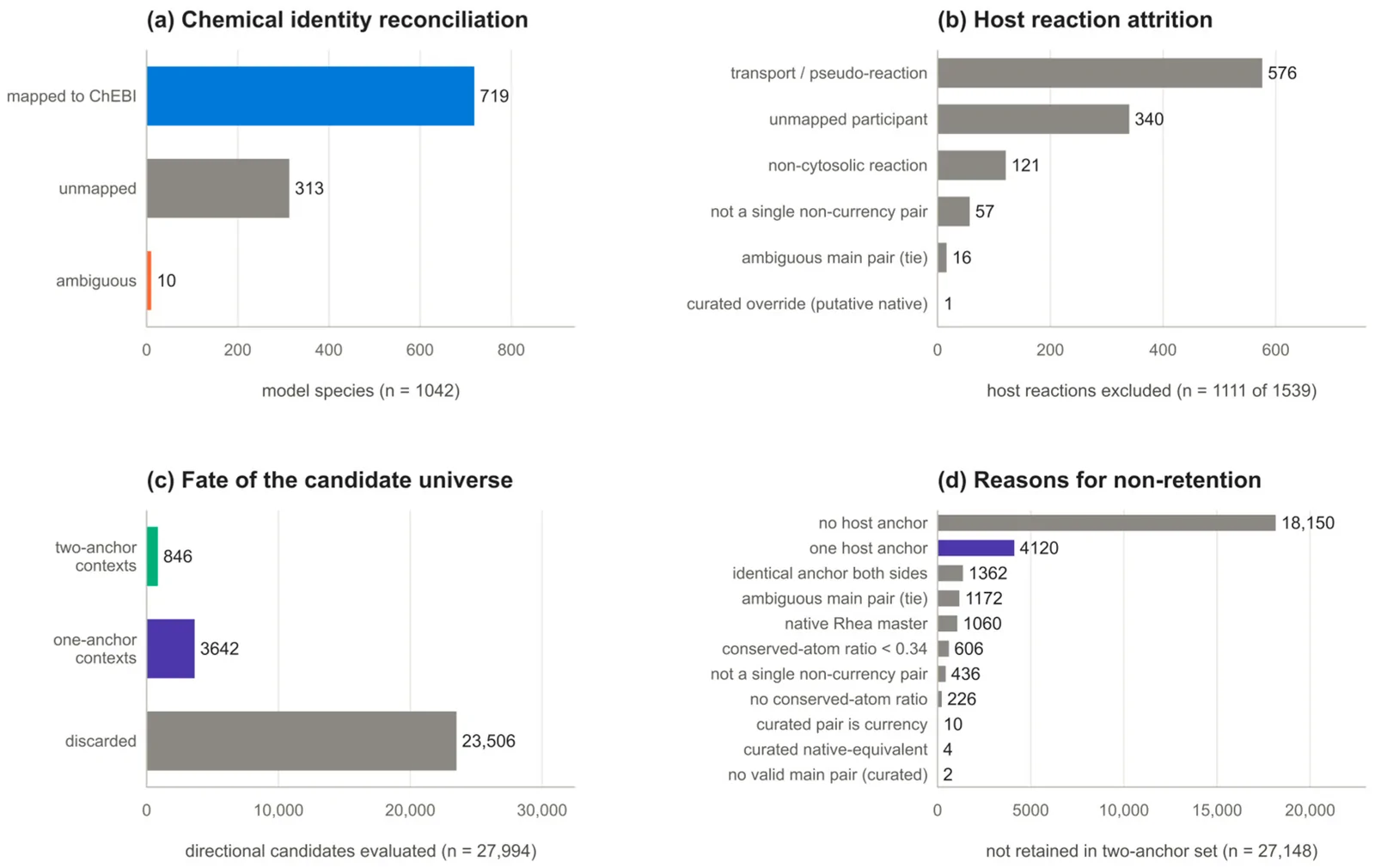
Generation of HESD records from the iCGB21FR model and Rhea release 141. The results of matching metabolites to ChEBI, building the model graph and selecting 846 variants with two and 3642 variants with one matched metabolite of the main pair are shown. Blue and orange denote mapped and ambiguous metabolites, respectively (**a**); green and purple denote two-host-anchor and one-host-anchor categories, respectively (**c**,**d**). Gray denotes the remaining mapping outcomes or exclusion categories.

The sequence by which the final selection was obtained is presented in Figure 3, and the reasons for excluding individual variants in Table S2–S4 and the Supplementary Materials.

The composition of HESD and the principal characteristics of its records are summarized in Table 1. An internal identifier links a particular reaction direction to the source equation in Rhea, to the metabolites of the main pair, to external identifiers, to the results of classification and validation, and to information on the associated proteins.

### 2.3. Topological Characteristics of the Reactions

**Figure 4:**
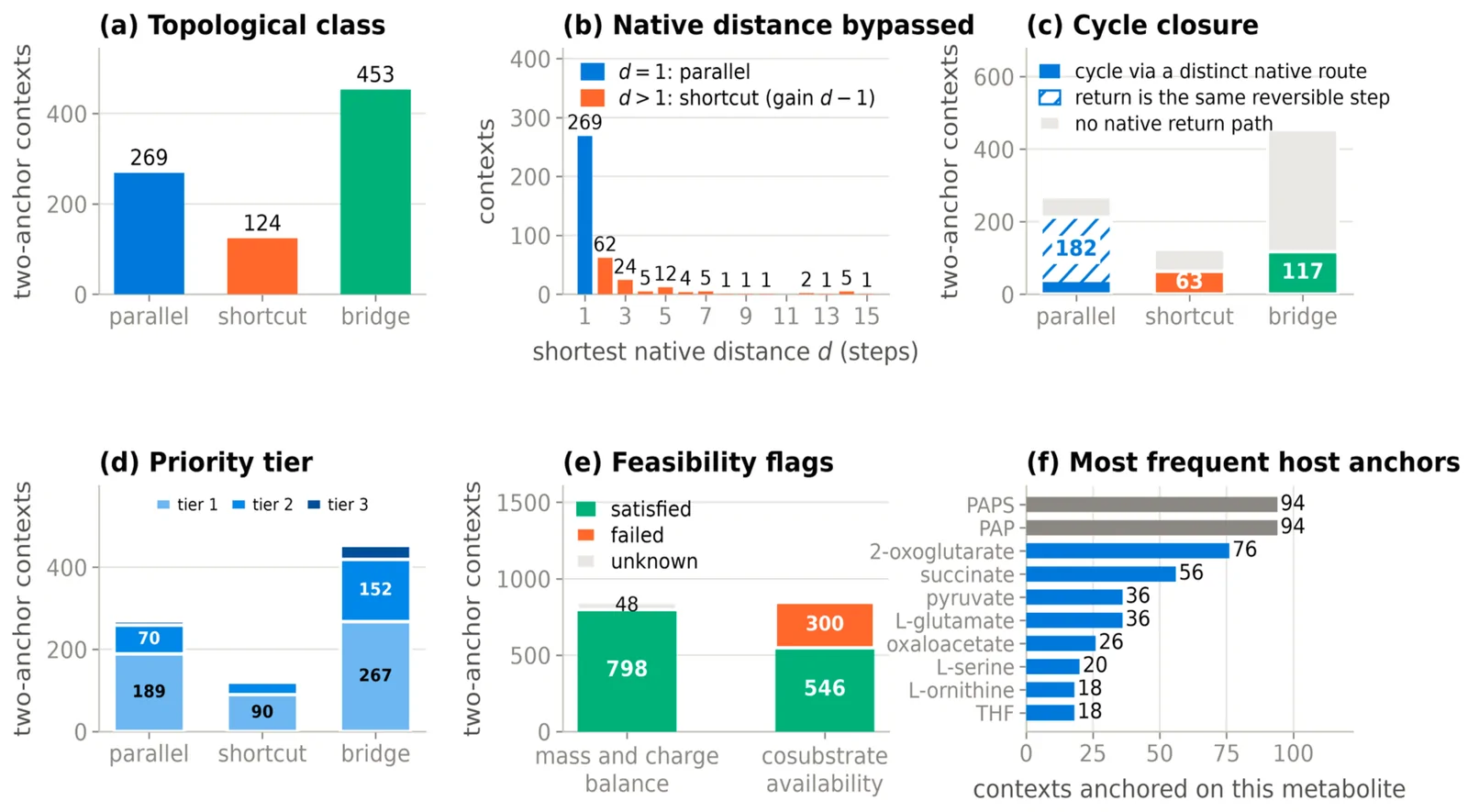
Results of the analysis of 846 reaction variants with two matched metabolites of the main pair: distribution by topological class (**a**), length of the paths replaced by path-shortening reactions (**b**), possibility of cycle formation (**c**), distribution by priority tier (**d**), results of the stoichiometric check (**e**) and the metabolites most frequently occurring in main pairs (**f**).

Among the 846 directed variants with two matched metabolites of the main pair, 269 parallel single-step reactions, 124 reactions shortening a path of the source model and 453 bridging reactions were identified (Figure 4a). These variants corresponded to 423 chemical conversions irrespective of direction. For 128 conversions, the forward and the reverse variant were assigned to different classes, because the presence and the length of a path in the directed graph depend on the direction of the reaction.

Reactions shortening a path of the source model most often replaced sequences of two or three steps; the maximum length of a replaced path was 15 steps (Figure 4b). The presence of a shorter representation of a reaction in HESD means a reduction in the number of steps in the graph, but does not in itself indicate an advantage of the reaction in terms of product yield, energy costs or kinetic characteristics.

For 393 directed reaction variants, a path from the product to the substrate existed in the source model, so that adding the corresponding reaction could close a cycle. This flag was set for 213 parallel single-step, 63 path-shortening and 117 bridging variants (Figure 4c). In 182 cases the reverse path corresponded to the direction of the reaction that is opposite to an already represented reversible reaction of the model. In the remaining 211 cases the cycle involved other iCGB21FR reactions.

When the graph was built taking into account the numerical FBC bounds of iCGB21FR, the number of two-sided variants was unchanged (846), but the distribution was 266 parallel single-step, 115 path-shortening and 465 bridging reactions. The class changed for 12 variants; the number of variants with a reverse path decreased from 393 to 381. The differences were caused by five reactions annotated as reversible but allowed by their bounds only in the reverse direction. The main tables describe the structural graph of the release, and the results of this check are given separately in Table S5.

The functional significance of a potential cycle is determined by the full stoichiometry of the reactions forming it and by the feasible flux distribution. In particular, counter-directed conversions of the same main pair that differ in their specificity towards nicotinamide cofactors can form a substrate cycle and redistribute reducing equivalents between NADH and NADPH without net consumption of the carbon substrate. The cycle-forming flag was therefore used as a basis for the subsequent stoichiometric and flux checks rather than as an independent assessment of the effect of a reaction.

### 2.4. Quality Control and Completeness of the Annotations

Elemental and charge balance was confirmed for 798 of the 846 variants; no unbalanced equations were found. For the remaining 48 variants the check was impossible because the molecular formulae of reaction participants were missing. Priority tier 1 comprised 546 variants with balanced equations, with cosubstrates either represented in the model or included in the list of auxiliary compounds, and with a confirmed or curated link between reaction and enzyme. Tier 2 comprised 252 balanced variants that did not satisfy one or both of the additional criteria, and tier 3 comprised 48 variants with incomplete information on the compounds (Figure 4d,e). The 2032 structural similarity hypotheses at evidence level E4, 316 with identical molecular formulae and 1716 structural analogues, are stored separately and are not included in exact matching or in the flux analysis. The sets with two and one matched metabolite are associated with 10,915 and 22,501 Swiss-Prot protein identifiers, respectively. For 500 of the 846 two-sided variants, at least one bacterial protein is available. EC numbers are given for 284 of the 423 chemical conversions with two matched metabolites and for 1032 of the 1821 conversions with one matched metabolite (Table S6–S8).

Main pairs were assigned unambiguously for 350 two-sided variants, by the conserved heavy-atom ratio for 490 and manually for six. The assignment method and the value of the ratio are stored in the database and are available for filtering; the manually assigned pairs and the grounds for their assignment are given in Table S9. The Fe^2+^ and Fe^3+^ ions are included in the list of auxiliary compounds. The catalogue contains 92 bridging variants with the main pair PAPS/PAP; full stoichiometric mapping is not available for them, so their flux was not assessed. When PAPS and PAP are included in the list of auxiliary compounds, 750 two-sided and 3648 one-sided variants are obtained; the two-sided set comprises 268 parallel single-step, 124 path-shortening and 358 bridging variants (Table S10).

Thirty fully mapped variants in iCGB21FR and 44 in iCW773 that have equivalent native reactions were annotated manually. For 17 and 29 of them, respectively, the direction of the reaction is already permitted by the flux bounds of the native reactions. The corresponding native reactions, proportionality coefficients and flux bounds are given in Table S11.

### 2.5. Interface and Search Capabilities

**Figure 5:**
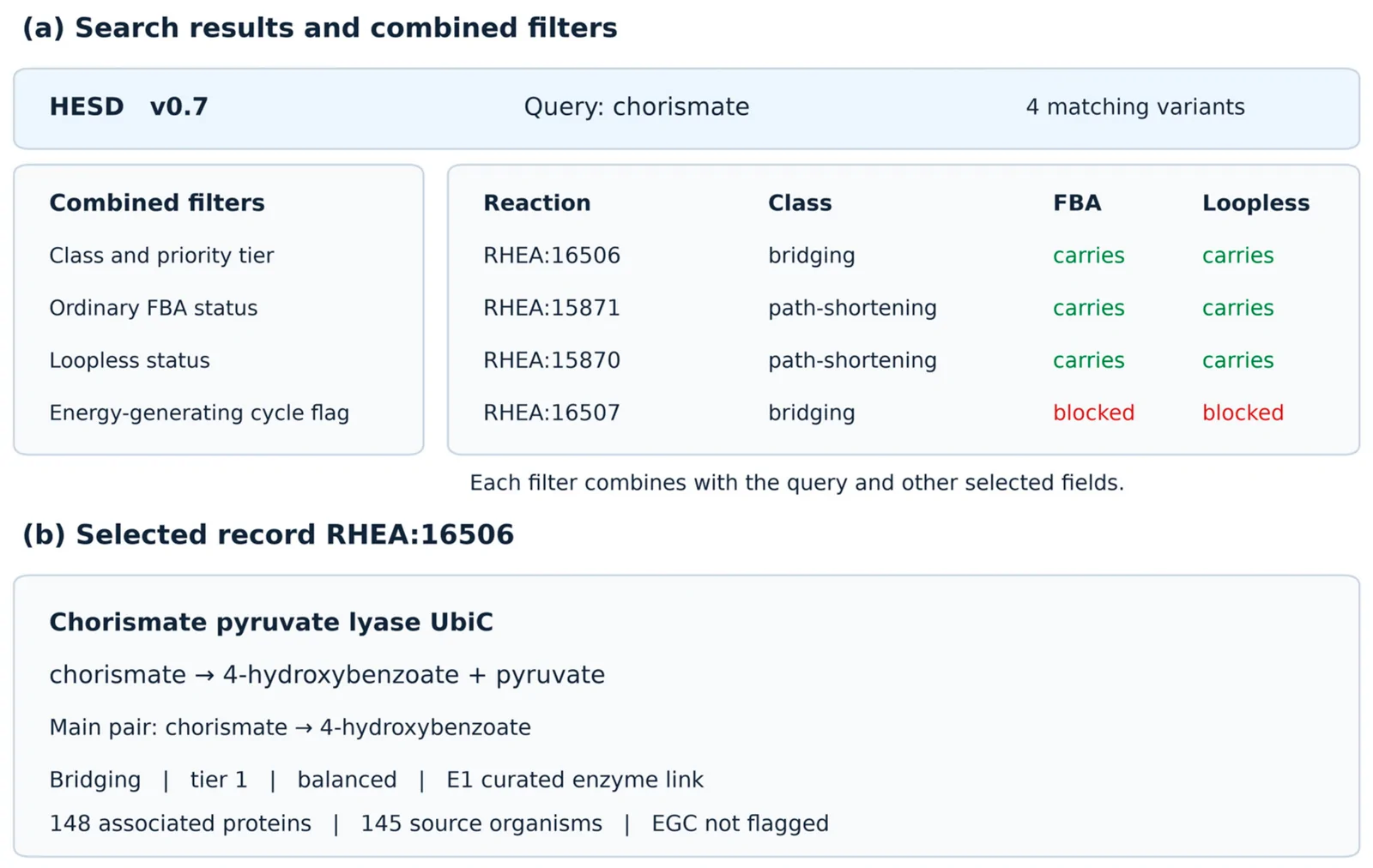
Schematic representation of selected HESD interface fields. The query “chorismate” and combined filters return four directed variants with their classes and ordinary and loopless flux statuses (**a**). The selected record RHEA:16506 shows the complete chorismate → 4-hydroxybenzoate + pyruvate conversion, main pair, evidence, priority and associated proteins (**b**). The schematic uses data from the current version of the database and simplifies the screen layout to keep the labels legible.

A web interface was developed for working with HESD (Figure 5), combining full-text search, filtering by record attributes and navigation between linked reactions, metabolites and proteins. A search can start from a chemical compound, a reaction, an EC number, or Rhea, ChEBI or Swiss-Prot identifiers. For each two-sided directed reaction variant, the interface displays the main pair, the topological class, the length of the metabolic path in the source model, the cycle-forming flag, the priority tier, the results of the stoichiometric check, and the associated enzymes and their source organisms. Flux feasibility, the energy-generating-cycle flag and the loopless status are filterable fields that combine with the other filters, and the result list is ordered so that variants shown to be unable to carry flux in the host model follow those that can. The underlying calculations are also presented in Tables S5 and S12–S23. The database is available at https://hesd.sysbio.ru (accessed on 19 October 2026).

Filters can be applied in combination. For example, a user may select reactions that use a given metabolite of the model and then restrict the selection to a particular topological class, to a confirmed balance, or to the presence of proteins of bacterial origin. Selected records can be exported in tabular and machine-readable formats for subsequent modeling and for compiling an experimental candidate list. The principal use scenarios of the interface are presented in Table 2.

### 2.6. Retrospective Validation on Published Examples

**Figure 6:**
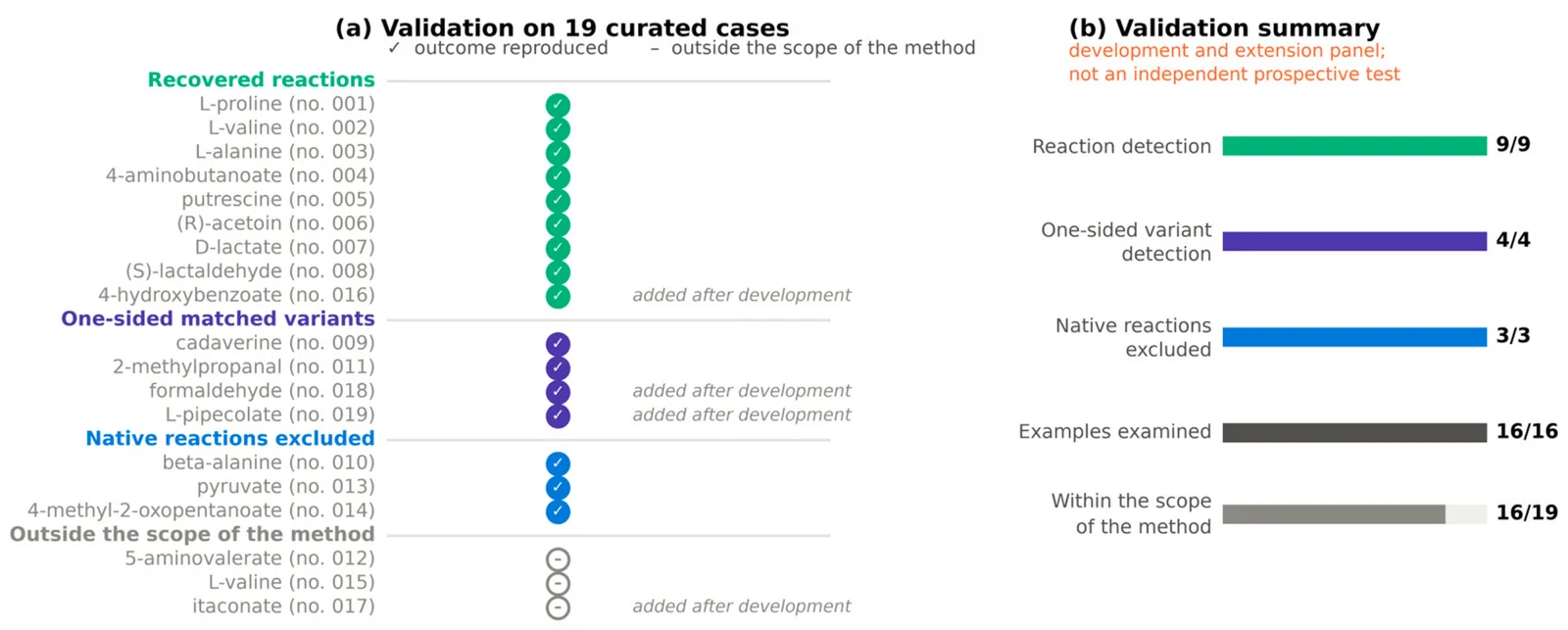
Retrospective validation of HESD on 19 curated cases comprising 18 published examples and one native-pathway control. The expected and obtained outcomes, the cases within the scope of the method and the reasons for excluding the remaining examples are shown, together with the separation of examples considered during the development of the system and after its completion.

For retrospective validation we assembled a panel of 19 curated cases: 18 published examples of *C. glutamicum* metabolic modification and one control based on a native pathway represented in iCGB21FR. The panel included interventions in amino acid biosynthesis pathways [4,5,6]. Sixteen examples fell within the scope of HESD. In nine cases the system identified the specified heterologous reaction, and in four it correctly identified a variant with one matched metabolite of the main pair. In a further three cases the reaction was known for *C. glutamicum* or was equivalent to a reaction already represented in iCGB21FR and was therefore excluded. Thus, the expected outcome was obtained for all 16 examples within the scope of the method (Figure 6).

The remaining three examples fell outside the scope of HESD for different reasons: one required the analysis of a multi-step heterologous pathway, the second concerned a change in the cofactor specificity of the IlvC enzyme, and in the third none of the metabolites of the main pair of a single reaction was matched to the model graph. The composition of the panel and the results for individual cases are given in Table S24 and in the Supplementary Materials.

Because a substantial part of the retrospective panel was used during the development of the system, this analysis characterizes the ability of HESD to reproduce known solutions but does not constitute an independent test of predictive power. After development had been completed, two further published interventions were examined: reaction RHEA:10297, catalyzed by NADP-dependent phosphorylating glyceraldehyde-3-phosphate dehydrogenase [7], and reaction RHEA:14670, catalyzed by non-phosphorylating NADP-dependent glyceraldehyde-3-phosphate dehydrogenase GapN [8]. HESD identified both reactions and assigned RHEA:10297 to the parallel single-step reactions and RHEA:14670 to the reactions shortening a path of the source model. Their functional effects are considered in Section 2.8.

In the analysis of a published route to L-valine, HESD identified the NADH-dependent reductive amination of 2-oxoisovalerate, RHEA:30765; however, the leucine dehydrogenase Q7SIB4 used experimentally was not among the proteins associated with this reaction. In the sources used, the reductive amination of 2-oxoisovalerate is not listed as the principal function of this enzyme. This example shows that identifying a suitable biochemical reaction and selecting a particular protein to carry it out are separate tasks; accounting for promiscuous activity requires additional literature analysis and experimental validation.

### 2.7. Flux Feasibility of Variants with Full Stoichiometry

**Figure 7:**
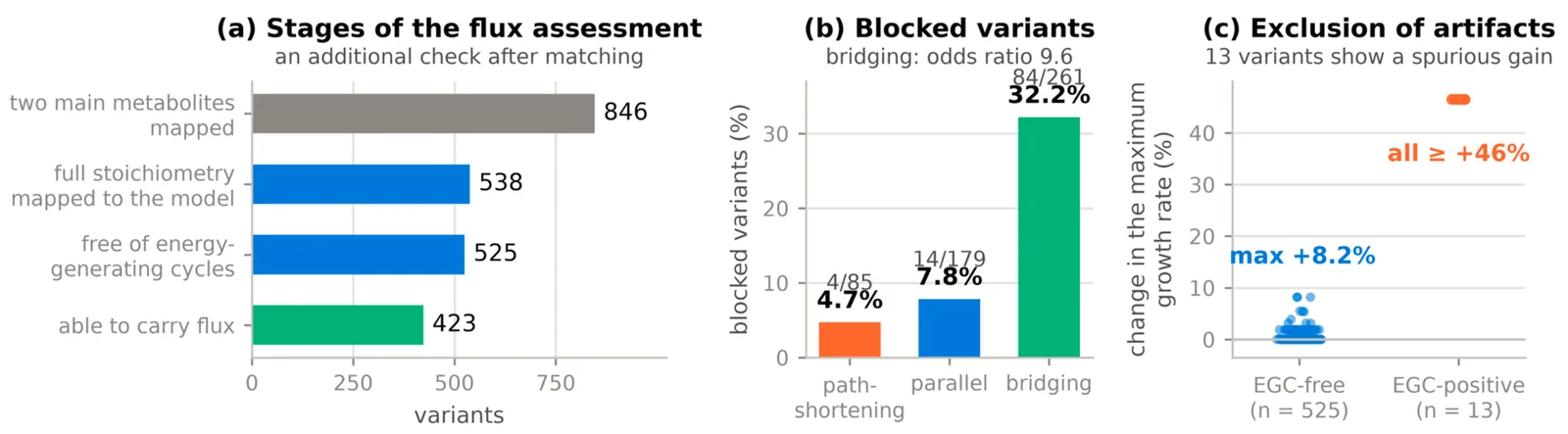
Flux assessment by ordinary FBA of variants with two matched metabolites of the main pair. The selection of variants with full stoichiometry (**a**), the proportion of blocked variants in the different topological classes (**b**) and the detection of internal energy-generating cycles (**c**) are shown. The results of the additional loopless check are given in Tables S26–S28.

Variants with two matched metabolites of the main pair were added individually to iCGB21FR, and the maximum possible absolute flux through the new reaction was calculated. Full stoichiometry could be transferred into the model for 538 of the 846 variants, for which all reaction participants had been matched (Figure 7a).

A preliminary check identified 13 variants that gave rise to internal energy-generating cycles (EGC-positive variants): the formation of ATP, NADH or NADPH persisted after the exchange reactions had been closed, without any input of external substrates. Their addition increased the maximum predicted growth rate by 46.5%, whereas among the remaining variants the largest increase was 8.2%. These 13 variants were excluded from the conservative flux assessment (Figure 7c).

In a separate loopless check, non-zero flux was confirmed for all 13 EGC-positive variants, with independent verification of the absence of internal cycles, but the global maximum was not proven (Table S25). The EGC warnings were retained, because the absence of cycles in a computed solution does not establish the physiological direction of a reaction.

In the conservative set of 525 variants, flux was possible for 423 under ordinary FBA, whereas 102 were blocked. The proportion of blocked variants was 4.7% among path-shortening reactions, 7.8% among parallel single-step reactions and 32.2% among bridging reactions (χ^2^(2) = 54.30; *p* = 1.62 × 10^−12^; Figure 7b). The odds ratio for a bridging reaction being blocked relative to a path-shortening reaction was 9.61. When the opposite directions of one conversion were combined, provided that both had been assessed and assigned to the same class, both directions were blocked for 23 of 97 bridging conversions, but for none of the 61 parallel single-step and 23 path-shortening conversions (χ^2^(2) = 22.82; *p* = 1.11 × 10^−5^).

All 84 bridging variants blocked under ordinary FBA had been assigned to priority tier 1 on the basis of stoichiometric correctness, cosubstrate availability and a confirmed link between reaction and enzyme. Meeting these criteria does not guarantee flux feasibility; the flux assessment therefore serves as an independent selection criterion.

Maximization of the flux with all boundary reactions closed revealed possible internal cycling for 134 of the 423 positive variants. After the loopless check, 117 of them retained non-zero flux in the medium considered, whereas 17 were blocked. In all 134 cases, the control flux with closed boundaries after the loopless constraints had been introduced was below 10^−6^ mmol gDW^−1^ h^−1^ (Tables S26 and S27).

For the remaining 289 positive variants, the maximum flux was retained after the cyclic components had been removed; the absence of cycles was confirmed by an independent check of the directions of all active internal reactions (Table S28). The conservative assessment thus comprised 406 variants with feasible flux and 119 blocked variants out of 525. Additional blocking was found for six parallel single-step, three path-shortening and eight bridging variants. Including the 13 separately assessed EGC-positive variants, the extended assessment comprises 419 variants with feasible flux and 119 blocked variants out of the 538 fully matched variants.

In the conservative set after the loopless check, the proportion of blocked variants was 11.2% among parallel single-step reactions, 8.2% among path-shortening reactions and 35.2% among bridging reactions (χ^2^(2) = 47.16; *p* = 5.74 × 10^−11^). With the same combination of opposite directions, both directions were blocked for 23 of 97 bridging, 1 of 61 parallel single-step and 0 of 23 path-shortening conversions (χ^2^(2) = 19.89; *p* = 4.80 × 10^−5^).

The robustness of the ordinary FBA results was tested under nine conditions: the glucose baseline and eight alternatives with fructose, gluconate, acetate or sucrose in place of glucose, oxygen limitation or deprivation, ammonium limitation and a fivefold lower carbon uptake limit. The maximum growth rate of the unmodified model ranged from 0.094 to 0.844 h^−1^. The set of blocked variants was the same in eight scenarios and differed by two variants when sucrose was supplied: 423 variants carried flux under all conditions, 100 under none, and the two sucrose-consuming variants only in the presence of this substrate. The association between topological class and blocking was retained in all scenarios; bridging reactions were blocked most frequently. These calculations tested whether non-zero flux was possible without a positive growth requirement or a target-product demand. The loopless check applies to the glucose baseline; the alternative media were assessed by ordinary FBA (Tables S29 and S30).

### 2.8. Examples of the Use of HESD

The possible applications of HESD are illustrated by reactions that differ in their position within the metabolic network and in the nature of their predicted effect. Adding the bridging reaction of chorismate pyruvate lyase UbiC (RHEA:16506) made the synthesis of 4-hydroxybenzoate from glucose possible. In the source model, the formation of this compound was not possible, whereas after the reaction had been added the maximum predicted flux was 7.17 mmol gDW^−1^ h^−1^. This example shows that a bridging reaction can confer on the model the ability to form a new product.

NADP-dependent phosphorylating glyceraldehyde-3-phosphate dehydrogenase (GapA) (RHEA:10297) represented an alternative to the single-step NAD-dependent reaction of iCGB21FR. Its addition increased the maximum predicted flux of L-lysine formation from 7.89 to 8.36 mmol gDW^−1^ h^−1^ through the additional formation of NADPH [7]. The GapN reaction (RHEA:14670), which replaces two consecutive glycolytic reactions by a single step, did not change the L-lysine flux under the initial conditions. After the principal routes of NADH/NADPH interconversion had been constrained, its addition increased the flux from 7.50 to 7.89 mmol gDW^−1^ h^−1^ [8]. A comparison of these examples shows that the same direction of change in the cofactor balance does not produce the same functional effect: the outcome depends on the position of the reaction within the network and on the available routes for redistributing reducing equivalents.

ArcE was selected as the prospective example because L-arginine is an established fermentation product of *C. glutamicum* and the reaction changes the energy and nitrogen requirements of its terminal biosynthetic route. The database shortlist required a path-shortening variant, complete stoichiometric mapping, loopless flux, absence of an EGC warning and a curated enzyme link. Seventy-eight directed variants satisfy these criteria in the current version of the database, all at evidence level E1. Table 3 compares ArcE with six alternatives. Path shortening alone does not confer a stoichiometric yield advantage; the net reaction and the model constraints must be examined.

As a prospective example, reaction RHEA:79573, catalyzed by ArcE [26], was investigated. In the calculation, its direction of L-arginine synthesis from L-citrulline was considered; in this direction it replaces the two consecutive steps of arginine synthesis from citrulline catalyzed by argininosuccinate synthase (ArgG) and argininosuccinate lyase (ArgH). Because the source model iCGB21FR lacked the reaction catalyzed by diaminopimelate dehydrogenase Ddh, which is known for *C. glutamicum* [27], this reaction was restored both in the control model and in the model with ArcE. After this correction, the addition of ArcE increased the maximum L-arginine formation flux at a minimum growth rate of 0.05 h^−1^ from 5.72 to 7.02 mmol gDW^−1^ h^−1^, that is, by 22.7%. Consequently, the positive predicted effect of ArcE was retained after a known gap in the source reconstruction had been eliminated.

The modeled ArcE route is L-citrulline + NH_4_^+^ + ATP → L-arginine + ADP + phosphate + H^+^. The native net route is L-citrulline + L-aspartate + ATP → L-arginine + fumarate + AMP + pyrophosphate + H^+^. ArcE uses ammonium directly and converts ATP to ADP; the native route uses aspartate and converts ATP to AMP plus pyrophosphate. With pyrophosphate hydrolysis included, the native route consumes two ATP equivalents. Neither net equation directly consumes NADH or NADPH. These stoichiometric differences provide a basis for interpreting the modeled advantage, although the contribution of each network constraint to the predicted flux increases remains to be quantified.

In *T. kodakarensis*, ArcE has been characterized in the opposite direction, forming L-citrulline and ATP from L-arginine [26]. For the modeled synthesis direction, the component-contribution estimate [29] is ΔG′° = +7.59 ± 3.33 kJ mol^−1^ at pH 7.5, ionic strength 0.25 M, pMg 3.0 and 298.15 K. Across the specified concentration endpoint combinations, ΔG′ ranges from −15.24 to +37.85 kJ mol^−1^ and is negative in 10 of 64 combinations. For the net ArgG/ArgH reaction without pyrophosphate hydrolysis, ΔG′° is +7.25 ± 3.28 kJ mol^−1^ and ΔG′ ranges from −61.25 to +9.52 kJ mol^−1^, negative in 126 of 128 combinations. Adding pyrophosphatase changes the standard estimate to −9.21 ± 3.28 kJ mol^−1^. The endpoint fractions describe the stated sensitivity design and are not probabilities of cellular feasibility. ArcE directionality depends jointly on ATP/ADP, ammonium, phosphate and arginine/citrulline. With citrulline 0.1 mM, arginine 1 mM and phosphate 4.472 mM, ΔG′ is −0.11 kJ mol^−1^ at ATP/ADP = 100 and ammonium 10 mM. The concentration ranges are sensitivity inputs, not measurements in *C. glutamicum*. The model-derived flux advantage therefore establishes a stoichiometric opportunity, while the energy estimate identifies concentration conditions affecting its direction (Tables S31 and S32 and Supplementary Section S19).

Ddh participates in the biosynthesis of *meso*-diaminopimelate, a lysine precursor, and is not a component of the ArcE reaction. Adding it to both compared versions of iCGB21FR served as a control for the completeness of the reconstruction at the imposed growth rate. In the source model without Ddh the effect of ArcE was also retained: the maximum L-arginine flux increased from 5.7186 to 7.0166 mmol gDW^−1^ h^−1^ (+22.7%; Table S18). The predicted advantage of ArcE is therefore not due to the introduction of Ddh.

The catalogue also contains the same conversion without an energy input: in RHEA:19599, catalyzed by arginine deiminase (EC 3.5.3.6), L-citrulline and ammonium give L-arginine and water. That variant is associated with 148 proteins, among them enzymes of the genus Corynebacterium, and by the criteria above it is the more accessible candidate. It was not selected because the amidation of the ureido group costs +37.2 kJ mol^−1^ on its own, so in the direction of L-arginine the variant reverses a hydrolysis, whereas the ArcE reaction couples this conversion to ATP hydrolysis. The catalogue reports both variants with their associated donors and flux assessment results; selecting between them requires an energetic assessment in addition to the stoichiometric criteria. Table 3 lists the candidates examined alongside RHEA:79573 and the reason each was not taken as the prospective example.

The examples considered confirm that topological classification characterizes the position of an added reaction relative to the existing paths but does not make it possible to establish in advance the magnitude, or even the existence, of a functional effect. Selecting a candidate requires the complete stoichiometric equation, a flux analysis under the specified conditions and a check of the completeness of the source model.

### 2.9. Sensitivity of the Results to the Choice of Host Model

For independent validation, the analysis was repeated with the model iCW773 of the same *C. glutamicum* ATCC 13032 strain [15]. Because some of the metabolites of iCW773 lacked ChEBI annotations, before the comparison we transferred ChEBI annotations to 78 metabolites that matched between the reconstructions by normalized identifier, compartment, molecular formula and charge; the stoichiometry and the flux bounds were not modified. With the original annotations, 710 reaction variants with two and 2296 variants with one metabolite of the main pair were identified in iCW773, and after harmonization of the annotations 966 and 2784 variants, respectively.

Of the 666 two-sided matched reaction variants shared by the two models, the main pair agreed in all cases and the topological class in 583 variants (87.5%; κ = 0.788). Consequently, the classification of most shared candidates is robust to the choice of reconstruction, whereas the quantitative composition of the classes remains model-dependent (Table 4).

Under ordinary FBA in iCW773, full stoichiometry could be incorporated for 640 reaction variants; 23 of them created internal energy-generating cycles, and 364 of the remaining 617 were able to carry a non-zero flux. The association between topological class and blocking was retained: the proportion of blocked variants was 13.6% among the path-shortening, 22.6% among the parallel single-step and 53.7% among the bridging reactions (χ^2^(2) = 66.23; *p* = 4.14 × 10^−15^). Among the 355 variants assessed for flux in both models, the status agreed for 259 (73.0%): 230 were able to carry flux in both models and 29 were blocked in both.

After an analogous loopless check, flux was retained in the conservative set of iCW773 for 343 of 617 variants, and 274 were blocked; 21 positive results of ordinary FBA were blocked in addition. All 23 initially EGC-positive variants also permitted a certified flux, so that the extended assessment without their prior exclusion amounts to 366 of 640. Among the 355 shared variants without an EGC warning, the statuses agreed for 263 (74.1%); among all 378 shared fully matched variants, including the EGC-positive ones, for 282 (74.6%; Table S33 and Table S34).

In iCW773, reaction RHEA:79573 likewise replaced the two steps catalyzed by ArgG and ArgH. With natively represented Ddh, the addition of ArcE increased the maximum L-arginine flux at a minimum growth rate of 0.05 h^−1^ from 7.73 to 8.70 mmol gDW^−1^ h^−1^ (+12.5%). The positive effect of ArcE is thus reproduced in both reconstructions, although its predicted magnitude differs. For the control and the modified model in each of the two reconstructions, solutions free of internal cycles were obtained that reach the same L-arginine maxima; the ArcE effects of +22.7% and +12.5% are retained after this check (Table S35).

Taking into account the zero FBC bounds of SPODM and FHL in the structural graph of iCW773 reduced the number of its reactions from 337 to 335 and the number of one-sided variants from 2784 to 2764. The class changed for one of the 966 two-sided variants (Table S5).

## 3. Discussion

In the present work, HESD is regarded as a database of directed variants of individual biochemical reactions matched to the metabolic network of *C. glutamicum*. Unlike resources that describe reactions, compounds or proteins independently of a particular organism model, HESD retains the characteristics that arise only after such matching: the presence and the length of a metabolic path of the source model between the main metabolites, the possibility of forming a reverse path, the mappability of the participants of the complete equation to the model, and the results of the associated flux analysis. This makes it possible to evaluate one and the same curated reaction as a potential intervention in a defined metabolic network.

Considering the forward and the reverse direction separately is essential for interpreting the database. For 128 of the 423 chemical conversions, the classification of the reaction depended on the direction. At the same time, for 640 of the 846 reaction variants the link to a Swiss-Prot protein was established without specifying a physiologically preferred direction. Consequently, the presence of an associated protein confirms a known catalytic function but does not determine the direction of the reaction in the cell, which depends on thermodynamic conditions, on the concentrations of the participants and on the cofactor composition.

The topological class also depends on the way the metabolic model is represented as a directed graph and on the rule used to select the main substrate–product pair. A bridging reaction therefore means the absence of a directed path between the selected metabolites in the graph used, but not the proven absence of the corresponding sequence of conversions in the cell. Storing the source pair, the complete equation and the parameters used to build the graph allows the user to verify the basis of the classification. A fixed list of auxiliary compounds does not take into account differences in the role of metabolites between reactions. Structural data from Rhea can be used to refine the assignment of main substrate–product pairs.

Analysis of the 17 additional blocking events in iCGB21FR revealed a variety of network constraints (Tables S36–S38). For RHEA:10982 and RHEA:10294, flux was restored when the uptake of D-serine and of N-acetylglucosamine, respectively, was allowed. In other cases an independent substrate source, a product sink, or a combination of the two was required. For RHEA:29045, flux feasibility depended on the availability of dTMP and dUMP, illustrating the need to consider the participants of the complete reaction equation alongside the main pair THF/mlTHF. The complete stoichiometric equations of RHEA:50705 and RHEA:50706 are equivalent to those of the native ALCD3/ALCD3ir reactions represented in the model. These correspondences show that selection of heterologous candidates based on Rhea annotations needs to be complemented by comparison of complete reaction equations.

Allowing the reverse direction of MMM2 restored flux for RHEA:27668, RHEA:61342 and RHEA:57494. The B12-dependent conversion of methylmalonyl-CoA into succinyl-CoA, demonstrated experimentally for *C. glutamicum* [30], provides grounds for revising this bound with regard to cofactor availability. At the same time, the model-based restoration of RHEA:10294 on GlcNAc cannot be transferred directly to the wild type: functional import of this sugar was achieved by expressing heterologous *nagE* [31]. The diagnostic changes to the medium, sinks and bounds were not introduced into the frozen models.

Quality control in HESD combines complementary characteristics. For 798 of the 846 variants the balance of chemical elements and charge was confirmed; however, the stoichiometric correctness of an individual equation does not guarantee that it can be incorporated into a steady-state flux distribution. This was demonstrated by the flux analysis: under ordinary FBA, 102 of the 525 variants examined were blocked, and after the loopless check 119, with bridging reactions blocked more often than reactions of the other two classes. In addition, 13 variants created internal cycles that permitted the formation of ATP, NADH or NADPH without any input of external substrates, and were excluded as artifacts of the combination of the added reaction with the structure of the model.

The prospective analysis of ArcE showed that the positive predicted effect of the reaction is retained after the restoration of Ddh, a known reaction of *C. glutamicum* that was absent from the source reconstruction. This result underlines the need to re-examine promising interventions after known model gaps that could change the baseline flux towards the target product have been eliminated.

Comparing the results obtained with the iCGB21FR model of *C. glutamicum* [14] with those obtained with iCW773 [15] made it possible to separate robust conclusions from characteristics that depend on the particular reconstruction. The agreement of the topological class for 87.5% of the shared variants and the persistence of the statistical association between class and flux feasibility confirm the reproducibility of the principal patterns. At the same time, the differences in the number of reaction variants in each class and the agreement of the loopless status for 74.1% of the shared candidates without an initial EGC warning and 74.6% when all shared fully mapped candidates are included show that the specific classification and the flux prediction are sensitive to the composition of the reactions, to their directions, to transport processes and to the model constraints. The persistence of the positive effect of introducing ArcE in iCW773 with native Ddh reinforces this example, but the differences in the relative flux increase (22.7 and 12.5%) do not allow the magnitude of the effect to be regarded as model-independent.

Information on the associated proteins extends the possibilities of moving from a reaction to an experimental candidate, but it does not replace the choice of a particular gene for expression. One conversion may be catalyzed by enzymes from different organisms that differ in their catalytic properties; an individual enzyme may possess several activities. Selecting a donor requires an additional assessment of the reaction direction, cofactor specificity, taxonomic proximity, intracellular localization and the properties of the protein that are relevant for its expression in *C. glutamicum*. Promiscuous activities may be absent from the curated record of the principal function, as was shown for leucine dehydrogenase Q7SIB4.

Donor selection is based on five criteria, applied in the following order: the smallest possible taxonomic distance between the donor organism and the host, which is stored for cellular donors with a recognized lineage and selectable as a facet (same genus, same family, same order, other actinobacteria, other bacteria, archaea, eukaryotes); agreement between the cofactor specificity of the donor enzyme and the cofactor pools of the host; the absence of requirements that *C. glutamicum* cannot meet, such as compartmentation, unusual metal centres or post-translational modification; thermal and pH optima compatible with cultivation; and a curated rather than an inferred link between the enzyme and the reaction. The resource supports the first and the last of these directly; the remaining three require assessment from the literature. Applied to the catalogue, the first criterion leaves 126 of the 846 two-sided variants with a donor no further than the order Mycobacteriales, 56 of which carry flux after the loopless check, and 188 variants with a donor within the actinobacteria, 92 of which carry flux. Among the 78 candidates that met the conditions of the prospective example, three have a donor of the genus Corynebacterium and nineteen a donor within the actinobacteria, whereas RHEA:79573 has a single curated protein from an archaeon; ArcE was selected as the example because its stoichiometric change directly affects the biosynthetic route to a product of industrial interest. The archaeal origin of the ArcE enzyme is a consideration for subsequent donor evaluation and expression design. Viral sources and unclassified lineages are retained as source records but are not assigned a cellular donor-proximity rank.

**Figure 8:**
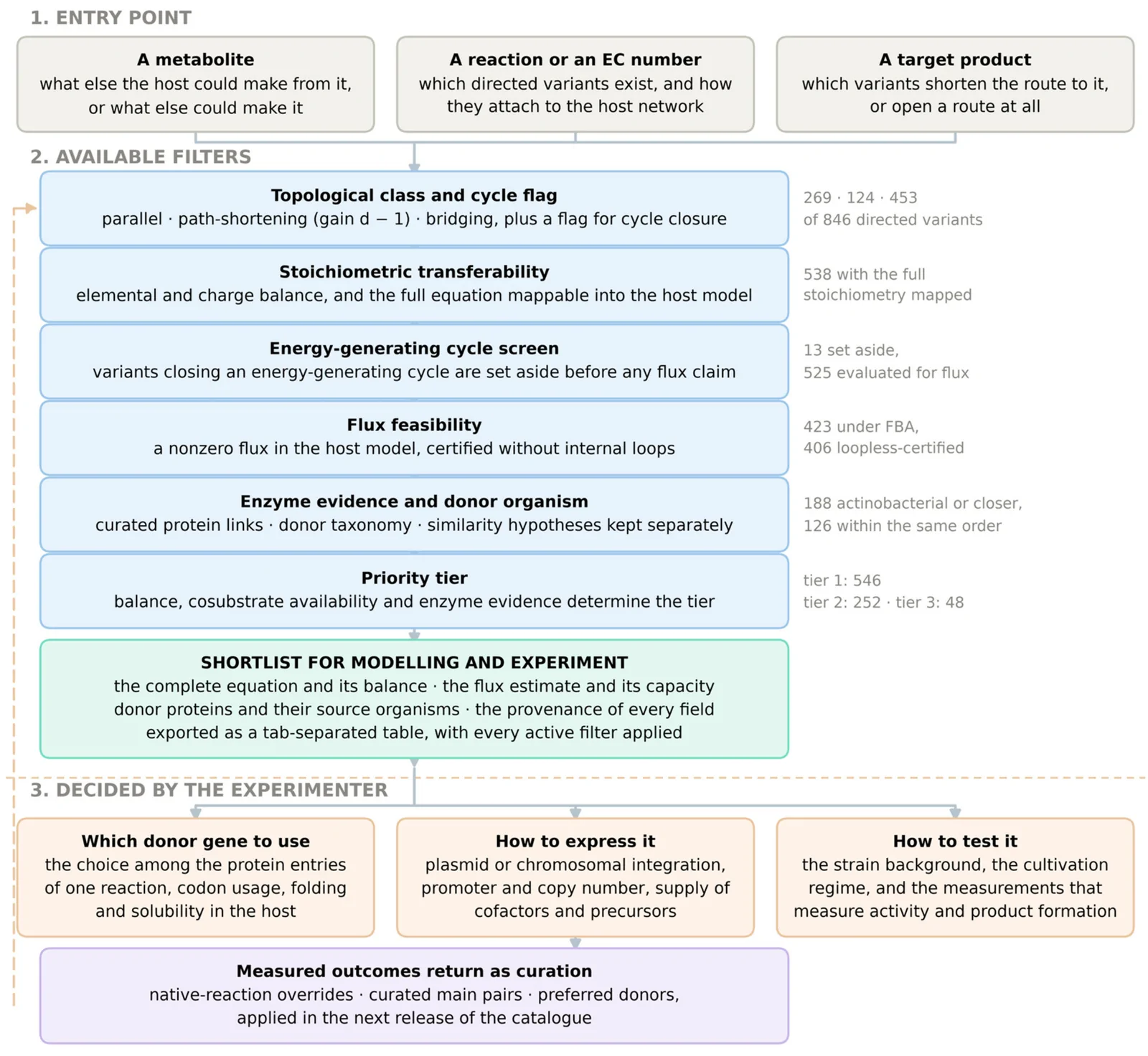
From a search in HESD to a strain-design decision. Available filters describe topology, stoichiometric mapping, EGC flags, flux status, enzyme evidence, donor taxonomy and priority tier. Counts refer to independent facets of the complete catalogue in the current version of the database and are not successive cumulative shortlist sizes. EGC screening and loopless certification do not establish physiological thermodynamic feasibility. The lower part shows donor selection, expression design and experimental testing, with measured outcomes feeding subsequent curation.

The HESD interface is designed for the stepwise narrowing of the search space. A user can start from a metabolite or a reaction, then take into account the topological class, the length of the metabolic path, the presence of a cycle, the priority tier and the origin of the enzyme, and directly filter the selection by ordinary and loopless flux status in the database. Such an organization does not tie the database to a single target product: reactions are first characterized relative to the entire model, and the specific product and cultivation conditions are defined at the next stage of modeling. Figure 8 sets out that sequence as a whole, from the entry point of a search to the decisions that the catalogue leaves to the experimenter.

HESD complements pathway-design tools and reaction knowledgebases (Table 5). It fixes the host reconstruction and records model-relative properties of retained individual directed reactions. The catalogue is specific to the chosen organism and reconstruction; it describes single-reaction contexts and uses stoichiometric and evidence criteria for prioritization. Product-specific route design and experimental strain evaluation provide subsequent analyses.

When interpreting the results, the limitations of the source data and of the methods must be taken into account. The determination of the main pair is based on gross composition and does not use explicit mapping of individual atoms. The molecular fingerprints used represent a compound by a set of features describing the local environment of atoms in the two-dimensional graph of chemical bonds. They make it possible to assess the similarity of molecular connectivity, but they do not account for stereochemistry, the reaction center or the catalytic mechanism; structural similarity hypotheses are therefore kept separate from exact matching. Full stoichiometry could be incorporated into the model for 538 of the 846 two-sided matched reaction variants. Flux balance analysis characterizes steady-state stoichiometric capabilities but does not account for kinetics, gene expression regulation, intracellular metabolite concentrations or thermodynamic constraints [9,32]. Removing internal cyclic fluxes narrows the set of feasible solutions, but without reaction energies and concentration ranges it does not establish physiological thermodynamic feasibility. For the same reason, a predicted increase in the maximum flux at a fixed growth rate is an upper bound on stoichiometric capability and not a predicted titre, yield or productivity. The relationship between the two is mediated by expression level, enzyme kinetics, regulation, by-product formation and process conditions, none of which the model represents. The ArcE results identify a stoichiometric advantage under the modeled conditions and motivate experimental testing of its effect on production.

HESD can be used for the preliminary selection of reactions in the development of producer strains, for comparing alternative ways of obtaining a given compound, for searching for possible bypass reactions and for analyzing the reasons why a candidate is blocked in the model. The complete content of the records and the information on their provenance make it possible to reanalyze the data when the host model or the annotation sources change. Further development is planned to include assessments of the thermodynamic feasibility of reactions, information on the kinetic properties of enzymes and a more complete description of promiscuous activities. We plan to extend HESD to additional microorganisms for which genome-scale metabolic models have been published, generating a separate host-specific catalogue for each selected reconstruction.

A separate direction is the integration of the HESD database with ANDSystem, a system previously developed by our group for the automated extraction of information from scientific publications and factual databases and for the reconstruction and analysis of associative gene networks [33,34]. We plan to match the metabolites and enzymes of the HESD database to the corresponding objects of ANDSystem in order to consider the identified reactions together with information on genes, proteins and their regulatory interactions. The stoichiometric and flux analysis will be retained as an independent component of the HESD computational system.

## 4. Materials and Methods

### 4.1. Study Design and Source Data

The host organism was *C. glutamicum* ATCC 13032, represented by the iCGB21FR model [14]. Candidates were selected on the basis of the Rhea annotations of the model and a list of curated exclusions. The absence of a corresponding annotation does not guarantee the absence of an equivalent complete equation; such cases were identified by comparing the full equations with the native reactions of the model (Table S11). The unit of analysis was the directed reaction variant; a variant with two matched main metabolites was considered two-sided and a variant with one matched main metabolite one-sided.

The analysis used the iCGB21FR model (MODEL2102050001), release 141 of the Rhea database, and ChEBI and UniProtKB/Swiss-Prot data downloaded on 17 August 2026. The taxonomic affiliation of the proteins was established using UniProtKB release 2026_03. For each source file, the version, the retrieval date, the license and the SHA-256 checksum were recorded. The complete list of sources is given in Table S1.

### 4.2. Chemical Identity Reconciliation and Selection of the Main Pair

Metabolites were matched by annotated ChEBI identifiers and by agreement of elemental composition and charge; in the case of several matches, a unique match of the normalized name was used [22,24]. Independent verification of stereocenters or structural keys was not performed in this procedure. Ambiguous correspondences were excluded from the exact analysis. The main pair was first determined after removing common auxiliary participants; when several candidates remained, the pair with the highest fraction of retained heavy atoms, not less than 0.34, was selected. The full stoichiometry and the way the pair was selected were stored in the database.

### 4.3. Construction of the Metabolic Model Graph and Classification of the Reactions

The cytosolic reactions of iCGB21FR were represented as a directed graph of main pairs; reactions with participants outside the cytosol were excluded. In the principal structural graph, a reverse edge was added when reversible=true in SBML; the numerical flux bounds were taken into account in the flux analysis. The sensitivity of the graph-based classification to the FBC bounds was checked separately, excluding blocked reactions and disallowed directions (Table S5). For a variant A → B, a breadth-first search algorithm determined the shortest path of the source model: at d = 1 the reaction was assigned to the parallel single-step reactions, at d > 1 to the path-shortening reactions, and in the absence of a path to the bridging reactions.

The presence of a path B → A was recorded as an indication of the possible formation of a cycle. If only one main metabolite was present in the model, the information on the variant was stored in HESD without determining a path of the source model between substrate and product.

In addition, the formation or consumption of metabolites represented in or absent from the source model, competition for a substrate and the formation of a cycle were recorded. These categories could overlap.

### 4.4. Feasibility Checks, Evidence Levels and Ranking

The full stoichiometry was used to check elemental and charge balance and the presence of the cosubstrates in the model. The cofactors of the heterologous and the native reaction acting on the same main pair were compared separately.

The evidence levels comprised experimentally confirmed reactions (E0), curated links to Swiss-Prot (E1), confirmed promiscuous activities (E2), predictions based on structure transformation rules (E3) and structural similarity hypotheses (E4). The confidence in the direction was recorded separately.

The priority tier was assigned on the basis of the balance, the cosubstrate availability and the evidence level. Detailed rules for assigning the priority tier and for ordering variants within a tier are given in the Supplementary methods.

For the E4 layer, a two-dimensional circular molecular fingerprint and the Tanimoto coefficient were computed. These hypotheses were stored separately and excluded from the exact counts.

### 4.5. Database Structure, Interface and Reproducibility

The HESD data-processing pipeline and web interface were implemented in Python 3.12, using SQLite 3 for data storage. In the HESD database, the source biological objects, the results of their matching, the characteristics of the directed reaction variants and the data provenance are kept separate. A stable variant identifier was computed from the host organism model, the reaction identifier, the direction and the matched main metabolites. For each record, external identifiers, the complete stoichiometric equation, the main pair, the topological class, the reverse path, the priority tier, the status of the checks and the associated proteins were stored.

Machine-readable data and exclusion reports were stored in SQLite, JSON and TSV formats. The search module supports queries by identifiers and text fields, the combination of filters, the inspection of record cards of linked objects and the export of a selection. The public web version of the HESD database is available at https://hesd.sysbio.ru (accessed on 19 October 2026). The database schema, the list of fields and the sequence of operations are described in detail in the Supplementary methods file.

HESD releases are versioned snapshots rebuilt from recorded source files and curation lists. Source adoption and curation changes trigger a rebuild, and the database is updated at least once a year. Each release records the source versions, retrieval dates and SHA-256 checksums. The distributed snapshots preserve earlier releases and allow analyses to be reproduced with the source inputs and rebuild commands used for that release.

### 4.6. Retrospective and Flux Validation

The retrospective panel comprised 19 curated cases, including 18 published examples and one native-pathway control, each with a predefined expected outcome. The detection of the reaction and the match to a particular donor protein were assessed separately.

Flux balance analysis was performed in COBRApy 0.32.1 [35], with GLPK accessed through optlang for linear programming. In the defined glucose medium, glucose uptake was constrained to 10 mmol gDW^−1^ h^−1^, the uptake of other organic compounds was excluded, biotin uptake was constrained to 0.001 mmol gDW^−1^ h^−1^ and ammonium uptake was allowed up to 100 mmol gDW^−1^ h^−1^.

RHEA:16506, RHEA:14670 and RHEA:10297 were added individually with their full stoichiometry. To model the formation of 4-hydroxybenzoate and L-lysine, demand reactions for the product were used; the routes of NADH/NADPH interconversion were constrained by blocking NADTRHD, ME1, ME2 and ALDD3y.

In the flux assessment, an individual directed variant was added with bounds [0; 1000] mmol gDW^−1^ h^−1^ without a positive lower bound on growth and was considered blocked when the maximum flux did not exceed 10^−6^ mmol gDW^−1^ h^−1^. As a separate control for internal cycling, the insertion flux was maximized with the exchange reactions, sinks and demand reactions closed. Energy-generating cycles were detected with closed exchange reactions by testing the possibility of forming ATP, NADH or NADPH [32]. The association between class and the presence of flux was assessed using Pearson’s χ^2^ test.

For the 134 EGC-negative variants with a positive flux under closed boundaries, a mixed-integer loopless FBA problem was solved [36]. The signs of the fluxes were related to metabolite potentials through G = Sᵀμ; these are technical variables for excluding internal cycles and not measured Gibbs energies. HiGHS 1.15.1, COBRApy 0.32.1, the same medium conditions and a zero lower bound on growth were used. For each solution, the consistency of the directions of all active internal reactions was verified separately. For the remaining 289 positive results, CycleFreeFlux [37] and the same independent verification were applied, confirming that the original maximum was preserved. All 17 additional blocking events persisted when the technical bound on the absolute value of G was increased from 10^3^ to 10^6^; the complete formulation and the numerical verification criteria are given in the Supplementary methods. The same check was extended to the 640 fully matched variants of iCW773 and, separately, to the 13 EGC-positive variants of iCGB21FR. A positive feasible solution with a verified mass balance and a direction certificate was considered proof that flux is possible even when the global maximum was not proven; such values were stored as lower bounds. A time limit with a zero solution was not considered proof of blocking. For the 21 additional blocking events in iCW773, the check at the technical bound G = 10^6^ gave the same result. Thermodynamic estimates were computed using equilibrator-api 0.8.1, equilibrator-cache 0.8.1, component-contribution 0.8.0 and equilibrator-cache-data 0.1.0 [29], as detailed in Supplementary Section S19.

For the prospective modeling of RHEA:79573 [26], the reaction of diaminopimelate dehydrogenase Ddh, which is known for *C. glutamicum* [27] and absent from the original iCGB21FR, was first added to both the control model and the model with ArcE. The Ddh reaction was added without introducing additional boundary sources of metabolites; the formation of its intracellular substrates was provided by the reactions of the source network and by the specified medium conditions, subject to the steady-state stoichiometric balance. The ArcE reaction with full stoichiometry was then introduced into the test model only, and the flux of a demand reaction for L-arginine was maximized at a lower bound on the growth rate of 0.05 h^−1^; the remaining medium constraints were as described above. The comparison was made against the control model with Ddh restored.

### 4.7. Sensitivity Analysis with Respect to the Choice of Host Model

The standardized version of iCW773 for *C. glutamicum* ATCC 13032 from BioModels (MODEL2110010001, version 2) was used as an independent reconstruction [15]. The principal analysis was performed with the iCGB21FR model [14], because it has a broader network coverage and more complete cross-annotations, which are required for matching to Rhea and ChEBI. To control for differences in the annotations, ChEBI identifiers absent from iCW773 were transferred from iCGB21FR only when the normalized metabolite identifier, the compartment, the molecular formula and the charge all matched. The stoichiometric coefficients, directions and bounds of the iCW773 reactions were not modified.

For iCW773, the preparation of the Rhea release 141 data, the matching of metabolites, the construction of the directed graph, the classification of directed variants, the check of the full stoichiometry, the detection of internal energy-generating cycles and the flux assessment were repeated. In both models, glucose and oxygen uptake were set to 10 and ammonium uptake to 100 mmol gDW^−1^ h^−1^, biotin uptake was constrained to 0.001 mmol gDW^−1^ h^−1^, and the uptake of the remaining carbon-containing compounds was closed, including CO_2_ in iCW773; the minimum growth rate in the ArcE modeling was set to 0.05 h^−1^. The consistency of the topological classes was assessed using Cohen’s κ coefficient, and the association between class and flux feasibility using Pearson’s χ^2^ test. The remaining mineral bounds were retained from the respective models. In the cross-model EGC control, all original boundary reactions were closed and ATP-, NADH- and NADPH-consuming reactions were added in turn; for iCGB21FR this protocol reproduced the same set of 13 exclusions as the control with closed exchanges. The causes of the 17 blocking events were investigated by 170 diagnostic interventions at a required insertion flux of 10^−4^ mmol gDW^−1^ h^−1^. Artificial sources and sinks served as a test of the network constraints and were not interpreted as existing physiological transport processes.

## 5. Conclusions

HESD links candidate reactions for heterologous introduction to the metabolic network of the *Corynebacterium glutamicum* ATCC 13032 strain and reports the assessment of the stoichiometric and flux feasibility of their addition. The candidate reactions are associated with 32,619 unique UniProtKB/Swiss-Prot protein records, of which 32,577 have taxonomic assignments from 3149 taxa, which allows the database to be used for selecting potential donor enzymes. HESD comprises 846 directed reaction variants for which both main metabolites are represented in the iCGB21FR model [14] and 3642 variants with one represented main metabolite. The HESD records and the associated Supplementary tables combine the chemical equation of the reaction, the topological characteristics, the results of the stoichiometric quality control and the flux check, data on the associated proteins and information on the sources of the original data.

The analysis showed that adding an individual reaction can enable the formation of a product inaccessible in the source model, can shorten a metabolic path represented in it, or can change the distribution of reducing equivalents between nicotinamide cofactors. At the same time, the topological class is associated with the flux feasibility of a reaction but does not in itself make it possible to determine the nature and the magnitude of its functional effect. After the additional check for internal cycles, flux remained feasible for 406 of 525 variants; 17 positive results of ordinary FBA depended on cycling.

Comparison with iCW773, another model of the same *C. glutamicum* strain [15], confirmed the robustness of the topological classification for 87.5% of the shared variants and the persistence of the positive effect of introducing the reaction catalyzed by ArcE with natively represented Ddh. The differences in the composition of the classes, in the flux status of the candidates and in the magnitude of the ArcE effect show that the quantitative estimates of HESD should be interpreted relative to the chosen host model. The conservative loopless assessment amounts to 406 of 525 variants for iCGB21FR and 343 of 617 for iCW773; the positive solutions for the original EGC variants are reported separately with their warnings retained.

The search and filtering tools of HESD connect a selected metabolite or reaction to candidate interventions and associated enzymes. The study provides computational candidate prioritization, including retrospective recovery, cross-model comparison, flux assessment and an ArcE thermodynamic sensitivity analysis. The records do not constitute experimentally validated strain designs. Further application requires candidate-specific modeling and experimental measurement of expression, activity and product formation. Integration with ANDSystem can add literature-derived regulatory information to this computational assessment. We plan to extend HESD to additional microorganisms for which genome-scale metabolic models have been published, generating a separate host-specific catalogue for each selected reconstruction.

## Figures and Tables

**Table 1:** Data composition and principal annotations of HESD.

HESD Component	Number	Content
Variants with two matched metabolites of the main pair	846	The path of the source model and the topological class can be determined
Variants with one matched metabolite of the main pair	3642	Enables the search for reactions forming a new product or incorporating a new substrate
Chemical conversions among variants with two matched metabolites	423	Unique conversions irrespective of direction
Associated Swiss-Prot identifiers	10,915/22,501	Number of distinct identifiers for variants with two/one matched metabolite
Structural similarity hypotheses	2032	Stored separately from the results of exact matching
Variants with a complete flux assessment	525	Assessed after matching the full stoichiometry and excluding internal energy-generating cycles; 406 retain feasible flux after the loopless check

**Table 2:** Principal tasks supported by the HESD search tools.

User Task	Information Used	Result
Finding reactions that consume or produce a selected metabolite	Model metabolite, ChEBI, role in the main pair, reaction direction	List of directed variants and possible products or substrates
Finding reactions that change the connectivity of the metabolic network	Topological class, path length, presence of a reverse path	Parallel, path-shortening and bridging variants
Selecting variants with the most complete support	Priority tier, balance, cosubstrate availability, evidence level of the reaction–enzyme link	Filtered list of records
Finding enzymes for a selected reaction	Rhea, EC, Swiss-Prot, taxonomic affiliation	Associated proteins and potential donor organisms
Restricting a selection to candidates able to carry flux	Full-stoichiometry mappability, flux status under ordinary and loopless FBA, the energy-generating-cycle flag	Candidate list filtered by flux feasibility in the host model
Using the results outside the interface	Selected fields and active filters	Export of the selected records

**Table 3:** Candidates that met the same selection conditions as RHEA:79573 in the current version of the database, and the reason each was not taken as the prospective example. All of them are path-shortening variants of priority tier 1 that carry flux after the loopless check and are linked to an enzyme at evidence level E1. ‘Steps bypassed’ is the length of the path of the source model that the variant replaces; ‘closest donor’ is the smallest taxonomic distance between a donor organism of the variant and *C. glutamicum*, with the number of associated proteins in parentheses.

Variant	Conversion (EC Number)	Steps Bypassed	Closest Donor (Proteins)	Why It Was Not Taken as the Prospective Example
RHEA:19599	L-citrulline → L-arginine (3.5.3.6)	2	same genus (148)	The same conversion without an energy input: in the direction of L-arginine it reverses a hydrolysis, the amidation costing +37.2 kJ mol^−1^ on its own
RHEA:20885	L-serine → 3-hydroxypyruvate (1.4.1.7)	14	actinobacteria (1)	The longest path among the compared EGC-free candidates, but the main product is an intermediate of serine metabolism and the variant closes a cycle with the host network
RHEA:11789	L-aspartate → oxaloacetate (1.4.1.21)	3	actinobacteria (54)	Replaces a step of central carbon metabolism; the predicted effect would concern the redox and nitrogen balance rather than a named product
RHEA:32289	(2S)-2-isopropylmalate → (2R,3S)-3-isopropylmalate (4.2.1.33)	2	same genus (1077)	Merges two steps of leucine biosynthesis without a net stoichiometric yield advantage; its effect depends on the imposed reaction bounds
RHEA:16514	(S)-lactate → acetate (1.13.12.4)	6	same order (1)	The product is an overflow metabolite rather than a target of strain development
RHEA:19206	dCTP → dUMP (3.5.4.30)	2	same genus (94)	Nucleotide salvage; the maximum insertion flux is 0.01 mmol gDW^−1^ h^−1^
RHEA:79573	L-citrulline → L-arginine (ArcE)	2	archaea (1)	Selected: L-arginine is an established product of *C. glutamicum* and the variant changes the net stoichiometry of the route it replaces

**Table 4:** Comparison of the HESD results obtained with iCGB21FR and iCW773.

Metric	iCGB21FR	iCW773
Variants with two metabolites of the main pair	846	966
Variants with one metabolite of the main pair	3642	2784
Parallel single-step	269	283
Path-shortening	124	76
Bridging	453	607
Variants with full stoichiometry	538	640
Internal energy-generating cycles	13	23
Variants with non-zero flux under ordinary FBA	423 of 525 (80.6%)	364 of 617 (59.0%)
Maximum L-arginine flux without ArcE	5.72	7.73
Maximum L-arginine flux with ArcE	7.02 (+22.7%)	8.70 (+12.5%)
Loopless FBA excluding the original EGC variants	406 of 525 (77.3%)	343 of 617 (55.6%)
Loopless flux including the EGC variants with the warning retained	419 of 538 (77.9%)	366 of 640 (57.2%)

**Table 5:** HESD and existing resources for the design of heterologous conversions.

Resource	Starting Point	What it Returns	What It Does Not Provide
Reaction and pathway resources including ATLASx and route-design methods [11,12,13]	A target compound	Known and predicted reactions or extracted routes; pathway-design methods provide route ranking	A product-independent survey of known single reactions against the whole network of a given host
Host-aware pathway design [10]	A target compound and a host model	Routes that the chosen host can support	Characterisation of reactions that do not lie on a route to the chosen target
Reaction knowledgebases: KEGG, Rhea [20,21]	A reaction or a compound	Curated equations, directions, enzymes and cross-references	The position of a reaction relative to the metabolic network of a particular organism
HESD (this work)	A host model	Retained directed reaction candidates assessed against the host model, with topology, balance, flux status and associated proteins	Multi-step routes, kinetics, regulation, and predictions of titre or yield

## Data Availability

HESD is accessible through a public web interface at https://hesd.sysbio.ru (accessed on 19 October 2026).

## Supplementary Materials

The following supporting information can be downloaded at https://www.mdpi.com/article/10.3390/ijms27219593/s1.
