# Supplementary material for: HESD: A Database of Candidate Reactions and Enzymes for Metabolic Engineering of Corynebacterium glutamicum
Source: Int J Mol Sci. 2026 Oct 27;27(21):9593. doi: 10.3390/ijms27219593 (PMC13763154; doi:10.3390/ijms27219593)
Supplement: Supplementary file 1 [file ijms-27-09593-s001.zip › HESD_Supplementary_Methods_and_Extended_Results.pdf]

Supplementary Methods and Extended Results

Supplement to the article "HESD: A Database of Candidate Reactions and Enzymes for Metabolic Engineering of *Corynebacterium glutamicum*"

This supplement describes the Rhea 141 × iCGB21FR set, the cross-model comparison with iCW773, and the flux calculations. The classes of the main catalogue refer to the structural graph built from the SBML reversibility attribute. Sensitivity to the numerical flux bounds is reported separately.

Section 14 reports a loopless FBA check: of the 134 variants with possible internal cycling, 117 retain flux and 17 are blocked. Taking into account the independent check of the remaining 289 positive variants, the result is 406 variants with feasible flux out of 525. The numbers 423 and 102 in Section 5 refer to ordinary FBA and are given for comparison of the two methods.

Sections 15 and 16 extend the analysis with a check of the original EGC-positive variants, a full loopless evaluation of iCW773, and an examination of the 17 blocking events. The conservative totals excluding the original EGC warnings are 406/525 for iCGB21FR and 343/617 for iCW773. When EGC-positive variants with certified flux are included, the extended totals are 419/538 and 366/640; the EGC warnings are nevertheless retained.

Row-level data are provided in the workbook HESD_Supplementary_Tables.xlsx. The present file preserves the rules used to build the database, the calculation conditions, the aggregated results, and the limits of interpretation; the table workbook contains the source records and the results for each variant.

1. Fixed Input Data

The host organism was *Corynebacterium glutamicum* ATCC 13032, represented by the genome-scale metabolic model (GEM) iCGB21FR (BioModels MODEL2102050001; 1042 metabolites and 1539 reactions). Fixed versions of Rhea 141, ChEBI, UniProtKB/Swiss-Prot, and data on the taxonomic origin of the linked proteins were used. The versions of the sources are given in Table S1.

We considered 13,997 Rhea chemical transformations that have links to curated Swiss-Prot proteins, in two orientations, for a total of 27,994 directed reaction variants. This is not the complete Rhea 141 reaction set. The separation into two directions reflects the directionality of path search in the metabolic model; the existence of an enzyme record for the underlying transformation does not by itself prove its physiological activity in both directions.

2. Metabolic Model Preparation and Compound Mapping

2.1. Chemical Identity

Among the ChEBI identifiers annotated in the SBML, those present in the frozen Rhea SDF and matching in elemental composition and charge were selected. When several matches occurred, a single match of the normalized name was accepted; any remaining ambiguity was excluded. Independent comparison of InChIKeys or stereocenters, and individual atom mapping, were not performed in this algorithm. Chemical identity is inherited from the annotated ChEBI entries and depends on the correctness of those annotations.

Unambiguous (one-to-one) ChEBI mappings were obtained for 719 model metabolites. After merging the entries of the same compound in different compartments, a set of 477 unique compounds represented in the cytosol was formed. Ambiguous mappings were not included in the exact mapping. The full mapping protocol and the reasons for exclusion are given in Table S2.

2.2. Main Substrate–Product Pair and the Native Reaction Graph

After excluding common auxiliary participants (currency metabolites) of the reactions, the main substrate–product pair was determined for each equation. If several admissible pairs remained, the fraction of conserved heavy atoms, calculated from the elemental composition, was used. The rule does not perform exact atom mapping and does not resolve stereochemical ambiguity. For the graph of native transformations, only cytosolic reactions were retained: the presence of a participant in the periplasm or in the extracellular space is grounds for exclusion, even if the chemical name coincides with that of a cytosolic metabolite.

The cytosolic graph retained 428 reactions with a main pair, connecting 366 distinct metabolites. Transport, pseudo-reactions and reactions with non-cytosolic participants were excluded; Table S3 lists the reasons for all host-reaction exclusions.

Stage	Count
iCGB21FR reactions	1539
Excluded from the cytosolic graph	1111
Reactions with a main pair retained	428
Graph metabolites	366
Rhea variants with both main metabolites present in the model	846
Rhea variants with one main metabolite present in the model	4120
Of these, connected to the cytosolic graph	3642

Of the 4120 directed candidates for which one main metabolite is represented in the model, 478 had no connection to the graph of the cytosolic reactions used and therefore did not enter the one-sided mapped set. The reasons for excluding all host and Rhea reactions are presented in Tables S3–S4.

3. Classification and Verification of the Equations

In the main release, the direction of an edge is set by the SBML equation, and the reverse edge is added when reversible=true. The numerical FBC bounds do not participate in this structural construction; they are taken into account in the flux model. A graph path may therefore include a direction that is forbidden by the model bounds. The reactions ALCD2y, HSDx, HSDy, LDH_L, and PPGKr in iCGB21FR have bounds [−1000; 0] although they are recorded as reversible. This case is examined separately in Section 10 and in Table S5.

For a variant A → B, a breadth-first search algorithm determined the length of the shortest directed native path d(A,B). At d = 1 the variant was assigned to the parallel single-step reactions; at d > 1, to the reactions shortening the native path by d − 1 steps; and in the absence of a path, to the bridging reactions. The reverse path B → A was recorded

independently as a structural cycle-forming flag. The class refers to a specific direction and to a specific version of the graph, and not to the reaction independently of the model.

Class or flag	Directed variants
Parallel	269
Path-shortening (native path)	124
Bridging	453
Total with both main metabolites mapped	846
Class differs between the two directions of a transformation	128 of 423
Native reverse path present	393

For 182 variants the reverse transformation was provided by the same reversible reaction of the model. For the remaining 211 variants the reverse path ran through other native reactions: 87 cycles had a total length of two steps, 62 of three, 24 of four, and 38 of no fewer than five steps. Among the 393 variants with a reverse path there were 213 parallel, 63 path-shortening, and 117 bridging reactions. The existence of a closed sequence does not indicate that it actually operates; establishing that requires analysis of the full stoichiometry and of the fluxes.

Elemental and charge balance was confirmed for 798 of the 846 variants. For 48 variants the check was impossible because the required chemical information was missing; among the equations that were checked, no unbalanced ones were detected. Priority tier 1 was assigned to 546 variants, tier 2 to 252, and tier 3 to 48. The hesd_score metric was used only for ordering within a tier, and not as a probability of successful expression.

3.1. Sensitivity to the Main-Pair Definition Rule

In release v0.7, 350 two-sided variants use a single non-auxiliary participant on each side, 490 use conserved-atom ranking and six use curated main-pair overrides. The single-pair branch does not apply the $R \geq 0.34$ threshold. The main-pair assignment rules and the curated assignments are described in Section 17 and Table S9.

4. Proteins and the Definition of "Heterologous"

Three manual nativity decisions were used to generate the candidates: Rhea master transformation 24368 was allowed as potentially heterologous under unconfirmed native activity; 15641 was excluded as confirmed native; 32271 was excluded as a sequence equivalent of IPMD and OMCDC. The identifiers, grounds, and references for these decisions are recorded in the curated native-reaction override list. The list of 34 auxiliary (currency) compounds and the five preferred donor mappings are part of the fixed project configuration.

HESD selects candidates on the basis of the Rhea annotations of the model and of the curated exclusions. Such a selection does not guarantee the absence of an equivalent full equation in the model or the absence of catalytic activity in *C. glutamicum*. Additionally identified native equivalents are flagged in Tables S12 and S38. A link to proteins of this species was found for 38 two-sided mapped and 64 one-sided mapped directed variants; a link oriented to the specific direction exists for only 5 and 4 variants, respectively. These records are retained with an explicit host-protein flag and require manual verification before any claim of biological heterologousness.

Overall, 10,915 unique Swiss-Prot identifiers are linked to the two-sided set and 22,501 to the one-sided set. The presence of an identifier is evidence of a curated link between a protein and a chemical transformation, but it is not a choice of donor gene. Selecting a donor additionally requires data on reaction direction, cofactor specificity, localization, taxonomic origin, and experimental activity.

5. Defined Glucose Medium and Flux Balance Analysis

The calculations were performed with COBRApy for iCGB21FR. In the deposited model, uptake of 4-hydroxybenzoate, urea, and biotin is allowed in addition to glucose; dividing the product flux by the glucose uptake rate alone in such a state does not give a yield on a single carbon substrate. In the scenario used here, the lower bounds of EX_34dhbz_e and EX_urea_e were set to zero. D-glucose uptake through EX_glc__D_e was limited to 10 mmol gDW^{-1} h^{-1}, biotin through EX_btn_e to 0.001, and ammonium through EX_nh4_e to 100 in the same units. Other sources of organic carbon were closed. The bounds of the remaining mineral exchanges were retained from the original model; for oxygen, −10 was used in the aerobic scenario and 0 in the absence of oxygen supply.

The full stoichiometry of the added reaction was matched only against cytosolic metabolite species. For each two-sided mapped variant, the maximum flux through the added reaction was optimized separately. The absence of a nonzero flux at a threshold of 10^{-6} mmol gDW^{-1} h^{-1} defined a blocking event. The result means stoichiometric admissibility in this model and medium; it does not prove cellular flux, product secretion, or physiological feasibility.

In the energy-generating cycle (EGC) check, the exchange reactions were closed, three demand reactions for ATP, NADH, and NADPH were added, and their fluxes were maximized in turn. A positive maximum of any of them above 10^{-6} led to exclusion of the variant; there were 13 such variants. The field egc_atp denotes the maximum of the three checks, and not of ATP alone. In this check all three test demand reactions were present simultaneously.

In a separate control, all original boundary reactions, including sinks and product removal reactions, were closed without adding the test demand reactions. The maximum flux through the insertion was positive for 147 of the 538 mapped variants: all 13 variants with energy-generating cycles and 134 of the 423 remaining variants with a nonzero flux. Consequently, for a portion of the insertions the flux can be sustained by an internal cycle without any external supply of substances. This result is not equivalent to energy generation and does not prove productive flux.

Metric	Result
Total two-sided variants	846
Full stoichiometry mapped to the cytosol	538
Excluded after the energy-generating cycle check	13
Evaluated by flux balance analysis	525
Nonzero flux possible	423
Blocked	102

Class	Evaluated	Blocked	Share, %

Class	Evaluated	Blocked	Share, %
Path-shortening	85	4	4.7
Parallel	179	14	7.8
Bridging	261	84	32.2

Pearson's test gives $\chi^2(2) = 54.30$, $p = 1.62 \times 10^{-12}$. The odds ratio for a bridging variant being blocked relative to a path-shortening variant is 9.61. Grouping both directions by the Rhea master identifier, and retaining conversions with both directions assessed in the same class, gives 23 of 97 bridging, 0 of 61 parallel and 0 of 23 path-shortening conversions blocked in both directions: $\chi^2(2) = 22.82$, $p = 1.11 \times 10^{-5}$.

6. Verification of Functional Effects on Target Products

In the case studies, the original and the modified models were compared under the same medium and the same constraints. For 4-hydroxybenzoate and L-lysine, demand reactions removing the product from the cytosol were added, in order to assess the maximum capacity for formation rather than an existing export route. The reactions RHEA:16506, RHEA:14670, and RHEA:10297 were included separately.

Intervention and condition	Target product	Before → after, mmol gDW^{-1} h^{-1}
UbiC, standard medium	4-hydroxybenzoate	0 → 7.17
NADP-GAPDH, standard medium	L-lysine	7.89 → 8.36
gapN, standard medium	L-lysine	7.89 → 7.89
gapN, NADH/NADPH transfer routes restricted	L-lysine	7.50 → 7.89

Under simple addition of an optional reaction, the optimum of the target product cannot mathematically deteriorate: the feasible solution with zero flux through it is retained. To investigate a potential negative side effect, a minimum flux through UbiC was imposed separately. At a flux of no less than 1 mmol gDW^{-1} h^{-1} the L-lysine maximum decreased from 7.89 to 7.47; at minimum fluxes of 0.5, 2, and 3 it was 7.68, 6.91, and 5.63, respectively. This is a model trade-off in the distribution of precursors between products under obligatory operation of the reaction, and not proof that expression of UbiC is harmful in the cell. All values are preserved in Table S16.

6.1. Counter-Directed Cofactor Cycles

For ten pairs, both orientations of the heterologous equation were examined, giving 20 variants in total (Table S42). For each of the two main metabolites m, compensation of the contributions of the two reactions was imposed: $S_{\text{het,m}} \times v_{\text{het}} + S_{\text{native,m}} \times v_{\text{native}} = 0$. The ratio of the coefficients must be the same for both main metabolites. Therefore, when one equation is reversed, the sign of the coupling between the fluxes changes. The constant condition $v_{\text{het}} + v_{\text{native}} = 0$ is correct only for identically oriented main pairs and is not used here.

Counter-directed flux is admissible for all 20 orientations in both networks tested. A value of 1000 indicates that the imposed upper bound was reached, and not a physiological cycle rate. The L-lysine and growth maxima were not changed: they are optimized after the constraint on counter-directed flux is released. In the constrained network, NADTRHD, ME1, ME2, and ALDD3y were set to zero; 10 orientations raised the L-lysine maximum from 7.50 to 8.36.

6.2. The ArcE Case Study

In the calculation, the L-arginine-synthesizing direction of RHEA:79573 was chosen, with bounds [0; 1000] mmol gDW^{-1} h^{-1}. This choice does not imply irreversibility of the enzymatic reaction: for *T. kodakarensis*, the opposite physiological direction, with formation of L-citrulline and ATP from L-arginine, has been described (Michimori et al., 2026; https://doi.org/10.3389/fmicb.2026.1761990). The ability of the enzyme to sustain the calculated direction in *C. glutamicum* is not proved by flux optimization.

The designations ArgG and ArgH refer to argininosuccinate synthetase and argininosuccinate lyase; their reaction identifiers in the model are ARGSS and ARGSL. Ddh participates in the formation of meso-diaminopimelate, a lysine precursor, and is not a component of the ArcE reaction. Restoring Ddh serves as a control of reconstruction completeness at the imposed growth rate. In the original iCGB21FR without Ddh, the L-arginine flux also increases upon addition of ArcE: 5.718562 → 7.016574 (+22.698%). Consequently, the effect of ArcE is not due to the introduction of Ddh.

ArcE from *Thermococcus kodakarensis* (Q5JHM5; RHEA:79573) was modeled by the reaction L-citrulline + $NH4^+$ + ATP → L-arginine + ADP + phosphate + H^+. In the graph it replaces ARGSS and ARGSL. To assess intracellular formation of L-arginine, a demand reaction for its removal was added; the lower bound on growth was set to 0.05 h^{-1}. Into both compared versions of iCGB21FR, the native Ddh missing from the reconstruction was introduced: tetrahydrodipicolinate + $NH4^+$ + NADPH + 2 H^+ → meso-diaminopimelate + $NADP^+$. No additional boundary sources of metabolites were introduced.

Upon addition of ArcE to the model containing Ddh, the L-arginine maximum increased from 5.720543 to 7.019003 mmol gDW^{-1} h^{-1} (+22.698%). The elemental and charge balance of ArcE was satisfied. Under closed bounds, the control maxima of ArcE and of the ATP/NADH/NADPH demand reactions were zero. The minimum ArcE flux at an L-arginine formation of no less than the optimum minus 10^{-6} was 7.033320; there was no flux through ARGSS and ARGSL in the selected pFBA solution. Table S17 also reports the original model and the sensitivity to the addition of balanced reactions associated with *C. glutamicum* proteins: in the latter scenario the effect at the same growth rate was 6.873532 → 7.990482 (+16.250%).

7. L-Valine Synthesis Case Study: Addition of a Parallel Reaction

In variants S3 and S4 the native transaminase IlvE was left active, while the NADH-dependent leucine dehydrogenase (reaction RHEA:30765) was added as a parallel reaction for the reductive amination of 2-ketoisovalerate. In variants S2 and S4 an NADH-dependent IlvC was imposed separately. The four variants differ in the set of available reactions, and not in a rigid choice of a single amination route. Consequently, fixed values of NADPH/NADH consumption for the “routes” cannot be regarded as the actual total fluxes of the optimal model.

In the defined glucose medium, the maximum calculated L-valine yield was 1.00 mol mol^{-1} glucose for all four variants and for both oxygen supply regimes. To compare the production effect in the absence of oxygen supply, the same minimum growth rate of 0.05 h^{-1} was used. In variant S1 the maximum L-valine flux was 7.48; in S2, S3, and S4 it was 9.64 mmol gDW^{-1} h^{-1}. Consequently, the addition of LeuDH raised the value relative to S1, but gave no additional gain beyond the effect of changing the cofactor specificity of IlvC under these conditions.

Case	IlvC	Amination reactions	LeuDH flux	L-valine
S1	NADPH	IlvE	0	7.48
S2	NADH	IlvE	0	9.64
S3	NADPH	IlvE + LeuDH	19.78	9.64
S4	NADH	IlvE + LeuDH	10.10	9.64

In one of the optimal flux distributions of S3, the native IlvE consumed L-valine at a rate of about 10.12 mmol gDW^{-1} h^{-1}, whereas the added LeuDH formed it at a rate of about 19.78. When consumption of L-valine through IlvE was forbidden, the maximum product flux did not change (9.64 mmol gDW^{-1} h^{-1}). Consequently, opposing fluxes through these reactions are possible but are not necessary for the calculated improvement. For S4, the flux of L-valine consumption through IlvE in the selected parsimonious solution was substantially smaller, about 0.44. Alternative optimal distributions without additional constraints are not excluded.

8. Retrospective Benchmark and Its Scope of Application

The retrospective benchmark panel included 19 curated cases: 18 published examples and one native-pathway control derived from iCGB21FR. Sixteen corresponded to the subject of the HESD analysis: nine individual reactions were detected, four variants were correctly assigned to the one-sided mapped set, and three native or equivalent reactions were excluded. The remaining three cases fall outside the scope of the method for different reasons: one required the construction of a multistep path, one represented a change in the specificity of the native IlvC, and in the third neither main metabolite of the single reaction was connected to the graph of the model. Fifteen cases were used during development and do not constitute an independent prospective panel.

HESD detected the chemical transformation RHEA:30765 but did not map to it the experimentally used leucine dehydrogenase Q7SIB4 on the basis of the curated links employed: for this protein the transformation is a side activity. Consequently, detection of a reaction and selection of a particular donor enzyme are distinct stages of the investigation.

9. Database Structure, Reproducibility, and Limits of Interpretation

HESD stores flux_assessed, egc_flagged, flux_feasible_fba, flux_feasible_loopless, flux_capacity and flux_maximum_proven for each two-sided variant. The flux capacity is the proven loopless maximum when available and a certified feasible lower bound otherwise. NULL distinguishes an unavailable assessment from a failed one. EGC-positive variants retain their warnings and have no ordinary-FBA verdict in the conservative assessment. The interface and exports expose these fields; Tables S12–S13 provide the corresponding row-level records.

The classification threshold is 10^{-6}; comparisons are performed before rounding, and solver error is not substituted by zero flux. The cycle-control results are presented in Tables S13 and S42.

The main limitations are model and annotation incompleteness, sensitivity of graph classification to the selected pair and compartment, absence of atom mapping and of complete stereochemical resolution, and absence of kinetics, enzyme-expression costs and regulatory constraints. The ArcE energy analysis in Section 19 is a targeted thermodynamic sensitivity, not a network-wide thermodynamic flux model. FBA optima are stoichiometric capacities under the stated constraints rather than measured cellular fluxes or product titers.

For subsequent expansion of the database, it is advisable to preserve the full rules of chemical object normalization, the model versions and exchange bounds, the decisions on candidate exclusion, the reaction-protein links, and the manual conclusions on nativity. It is precisely this information that is required for reuse when thermodynamic, kinetic, and enzyme-constrained models are added.

10. Cross-Model Sensitivity and Flux Bounds

The independent reconstruction iCW773 was taken from BioModels MODEL2110010001, version 2: 1207 reactions, 951 metabolites and 773 genes. For 78 metabolites, the missing ChEBI annotations were transferred from iCGB21FR when the normalized identifier, the compartment, the molecular formula and the charge matched simultaneously (Table S45). The stoichiometry and the flux bounds were not modified by the transfer. With the original annotations, 229 graph reactions, 710 two-sided and 2296 one-sided variants were obtained; after reconciliation of the annotations, 337, 966 and 2784, respectively.

Metric	iCGB21FR	iCW773
Two-sided / one-sided variants	846 / 3642	966 / 2784
Parallel / path-shortening / bridging	269 / 124 / 453	283 / 76 / 607
Full stoichiometry / EGC exclusions	538 / 13	640 / 23
Non-zero flux / evaluated	423 / 525	364 / 617
L-arginine without / with ArcE at a growth rate of ≥0.05 h^{-1}	5.7205 / 7.0190	7.7319 / 8.6970

Among the 666 shared two-sided variants, the main substrate–product pair agreed in all cases; the class agreed for 583 (87.538%; Cohen's κ 0.787574). Among the 355 variants evaluated in both models after the exclusion of the energy-generating cycles (EGCs), the flux status agreed for 259 (72.958%): 230 were able to carry flux and 29 were blocked in both models. For 85 variants flux was feasible only in iCGB21FR, and for 11 only in iCW773. The row-level results and the transitions are presented in Tables S18–S23 and S46–S47.

In both models, the uptake limits were 10 for glucose and O_2, 100 for ammonium and 0.001 mmol gDW^{-1} h^{-1} for biotin. The remaining exchanges of carbon-containing compounds were closed; in iCW773 this includes CO_2. The remaining mineral bounds are retained from the respective reconstruction and do not coincide completely. In the cross-model EGC control, all original boundary reactions were closed and test consuming reactions were added in turn. This protocol differs from the exchange-based control of Table S13, but for iCGB21FR it yields

the same set of 13 exclusions. In iCW773, Ddh is already present as DAPDH. The ArcE effect equals +12.482%; the minimum ArcE flux near the optimum equals 8.707356.

The graph analysis was repeated separately taking into account the FBC bounds of the frozen models. In iCGB21FR, five reactions are allowed only in the reverse direction. Taking them into account preserves 846 and 3642 variants, but yields 266 parallel, 115 path-shortening and 465 bridging reactions; the class changes for 12 variants, and at least one of the class, path or cycle attributes changes for 24. The number of variants with a reverse path decreases from 393 to 381. In iCW773, excluding the blocked SPODM and FHL reduces the graph from 337 to 335 reactions and the one-sided catalogue from 2784 to 2764 variants; one two-sided variant, RHEA:21325, moves from parallel to bridging. The main release and this sensitivity analysis are kept separate in Table S5; in both cases the flux calculations take the FBC bounds into account.

11. Priority and Structural Similarity Rules

The list of auxiliary participants is applied irrespective of the biological role of a compound in a particular reaction. Consequently, reactions whose main substrate and main product are both excluded by this list — for example, the interconversions of NAD^+ and $NADP^+$ involving NAD kinase — are not assigned a main substrate–product pair and are not included in the exact catalogue. The replacement of a native enzyme by a more efficient heterologous enzyme that retains the same conversion, and the differences in their kinetic properties, are likewise not evaluated by the current algorithm. These limitations do not deny the biotechnological significance of such interventions.

When several main pairs were possible, `R = Σ_(e≠H) min(n_(e,A), n_(e,B)) / max(Σ_(e≠H) n_(e,A), Σ_(e≠H) n_(e,B))` was used. A pair was retained when $R \geq 0.34$ and no equal maximum was present within a tolerance of 10^{-9}. The rule of a single non-auxiliary compound on each side is applied first; for such an unambiguous pair the R threshold is not required. The method uses elemental composition and not atom mapping.

Priority tier 1: a positive elemental and charge balance is known, all additional participants are either represented in the model or included in the list of auxiliary participants, and the evidence level is E0 or E1. Priority tier 2: the balance is positive, but the additional conditions of tier 1 are not met, with evidence levels E0–E2. Priority tier 3: the balance is undetermined, with evidence levels E0–E2. Priority tier 4: at least one balance is negative, or the evidence level is weaker than E2. The automatic assumption that an auxiliary participant is available is a ranking rule and not the result of a check of the complete matching or of flux.

Within a tier, records are sorted by decreasing `hesd_score` = 0.40F + 0.35E + 0.25T, and then by the stable ID. T = 1−1/d for path-shortening reactions, 0.6 for bridging reactions and 0.1 for parallel single-step reactions. E takes the values 1.0; 0.8; 0.6; 0.4; 0.2 for E0–E4 and 0.1 for an unknown level. The base F equals 1 when both balances are positive, 0.25 when one is positive and the other negative, 0 when both are negative, and 0.5 when the balance is unknown; it is multiplied by 1, 0.6 or 0.85 when the availability of the additional participants is positive, negative or unknown, respectively. These weights are fixed and do not estimate the probability of experimental success.

For E4, an in-house deterministic circular fingerprint `hesd-circular-fp/v1` of radius 2 computed from V2000 connection tables is used, and not the RDKit ECFP. The Tanimoto

coefficient equals |F_A∩F_B|/|F_A∪F_B|; the threshold is 0.7. For each source one-sided candidate, at most three of the most similar host metabolites were retained. The fingerprint does not encode stereochemistry. The `isomer` label denotes agreement of the molecular formulae with differing identifiers, and not a proven interchangeability of the substrates. All 2032 E4 hypotheses are held separately in Table S40.

12. Units and Reading Conventions of the Calculations

Fluxes and the maximum rates of product removal and of uptake are expressed in mmol gDW^{-1} h^{-1}, and the specific growth rate in h^{-1}. A positive flux through a demand reaction indicates the possibility of intracellular formation, but not the existence of transport out of the cell. For the valine tables, the yield is divided by the specified glucose uptake limit of 10; the carbon yield additionally takes into account the 5 carbon atoms in valine and the 6 in glucose. Biotin remains a minor additional source of carbon.

In Tables S15 and S44, the fields `pathway_NADPH_per_val`, `pathway_NADH_per_val` and `net_transhydrogenation_per_val` are the formal costs of the selected route and not the actual integral fluxes of the network. `growth_at_that_point_h-1` refers to the condition of 50% of the maximum growth of the given variant, whereas the comparison at the common bound of 0.05 is stored separately. In Table S44, NADTRHD and ME2 were blocked; this is a different sensitivity analysis from the blocking of four reactions in Tables S14 and S42. An empty cell means the absence of an assessment or non-applicability, and not zero.

13. Contents of the Supplementary Table Workbook

Sheets	Contents
S0–S5	Table of contents, sources, metabolite matching, the exclusion protocol and sensitivity to the bounds.
S6–S8	EC numbers and data on donor proteins.
S9–S11	Curated main-pair assignments, PAPS/PAP main-pair sensitivity and full-equation native equivalence.
S12–S13	The two-sided set and the complete flux analysis.
S14–S17	Product examples, L-valine, the UbiC–L-lysine trade-off and ArcE profiles.
S18–S23	The cross-model comparison: summaries, transitions of classes and of flux statuses, row-level calculations and ArcE.
S24	The retrospective benchmark panel.
S25–S28	EGC re-evaluation, the loopless check of 134 variants, examples of cyclic fluxes and the confirmation of the remaining 289 positive results.
S29–S32	Medium scenarios, per-variant robustness, thermodynamic estimates and ArcE concentration sensitivity.
S33–S35	The loopless analysis of iCW773, the comparison of the models and the ArcE control.
S36–S38	The causes of blocking, diagnostic probes and native equivalents.
S39–S47	The one-sided set, similarity, the native graph, cofactor cycles, units and reading conventions, the constrained valine scenario, annotation transfer and the iCW773 catalogues.

14. Verification of Internal Cycles by Loopless FBA

The purpose of the check was to determine whether each of the 134 EGC-negative insertions permits flux outside an internal, stoichiometrically closed cycle. All 134 insertions were introduced individually into iCGB21FR with their full stoichiometry and bounds [0; 1000] mmol gDW^{-1} h^{-1}. The medium, the oxygen limit of 10 and the zero lower bound on growth were identical to those of the main census. A repeated ordinary FBA reproduced the original maxima.

14.1. Problem Formulation and Independent Verification of the Solutions

A mixed-integer formulation following the loopless COBRA principle (Schellenberger et al., 2011; https://doi.org/10.1016/j.bpj.2010.12.3707) was used in the equivalent representation `G = S_int^T μ`. For potentially cyclic internal reactions, a binary variable a links the sign of the flux to the opposite sign of G: `−M_r(1−a) ≤ v_r ≤ M_r a`; `1 ≤ G_r + (B+1)a ≤ B`. `M_r` is taken from the actual bounds of the corresponding reaction, and `B = 1000`. The potentials μ are free. They and G serve only to verify the consistency of the directions and are not measured Gibbs energies.

The preliminary list of potentially cyclic reactions was obtained with a threshold of 10^{-7} and five stopping checks. All 134 open and 134 closed problems terminated with a proven optimum and a zero MILP gap. Results obtained under the time limit were not counted as blocked.

Each flux distribution obtained was verified by an independent linear problem covering all active internal reactions, including those not present in the preliminary list: `sign(v_r) × S_r^T μ ≤ −1` when `|v_r| > 10⁻⁷`. Such a certificate was found for all 134 solutions. This confirms the absence of internal cycles in the entire active set and not only in the preselected reactions. When the maximum is zero, the weaker problem with constraints on a subset of the reactions already proves that the full loopless problem is blocked.

The maximum residual of the steady-state mass balance is 7.25×10^{-10}, and the maximum constraint violation is 9.23×10^{-10}. In all closed controls, the insertion flux is below the threshold of 10^{-6}; the largest absolute residual numerical value is 1.14×10^{-8}. All 17 additional blocking events persisted at `B = 10⁶`, which rules out any dependence of this list on the chosen technical bound of 1000 within the range tested.

14.2. Results for the 134 Variants

Class	Assessed	Flux retained	Blocked
Parallel single-step	69	63	6
Path-shortening	20	17	3
Bridging	45	37	8
Total	134	117	17

The maximum flux decreased for all 134 variants. For the 117 variants in which flux was retained, the median ratio of the loopless maximum to the ordinary maximum was 0.0533 (median decrease 94.7%). None of the 134 should be excluded merely because it is able to

form a cycle: 117 permit a non-zero flux outside such a cycle as well. The seventeen blocked variants are listed row by row in Table S26.

Table S27 gives one closed-loop flux for each insertion, found by minimizing the total absolute flux with the bounds closed and the insertion rate fixed, normalized to unit flux of the insertion. The witnesses comprise 2–6 reactions: 17 with two reactions, 73 with three reactions, 19 each with four and five reactions, and 6 with six reactions. These are examples confirming that a cycle is possible; they are neither an enumeration of all cycles nor measured physiological rates.

14.3. Control of the Remaining Variants and Overall Statistics

For the remaining 289 positive results of ordinary FBA, CycleFreeFlux (Desouki et al., 2015; https://doi.org/10.1093/bioinformatics/btv096) was applied with the original objective function and the exchange fluxes retained. In all cases the same insertion maximum was reached and the independent check of the signs of all active internal reactions was passed. A cycle-free feasible solution reaching the ordinary upper limit was thus constructed for these variants, which confirms that this limit is optimal for loopless FBA as well (Table S28).

Class	Flux feasible	Blocked	Blocked, %
Parallel single-step	159	20	11.2
Path-shortening	78	7	8.2
Bridging	169	92	35.2
Total	406	119	22.7

The summary for the 525 variants after the original EGC filtering is as follows: 406 permit flux (77.3%) and 119 are blocked, 102 of them already under ordinary FBA and 17 additionally under loopless FBA. The association between class and blocking is retained: $\chi^2(2) = 47.16$; $p = 5.74 \times 10^{-11}$. After combining the two directions of one conversion with a matching class, both directions are blocked for 23 of 97 bridging, 1 of 61 parallel single-step and 0 of 23 path-shortening conversions; $\chi^2(2) = 19.89$; $p = 4.80 \times 10^{-5}$.

The denominator of 525 in this section retains the original exclusion of the 13 EGC-positive insertions of iCGB21FR. Their separate re-evaluation and the extension of the analysis to iCW773 are presented in Section 15.

The absence of internal cycles does not prove that a reaction is feasible at real concentrations, nor does it prove its physiological direction, its kinetic efficiency or the activity of the enzyme in the cell.

15. EGC Variants and Loopless Comparison of the Models

All 13 originally EGC-positive variants of iCGB21FR and all 640 fully matched variants of iCW773, including the 23 with an original EGC warning, were re-checked. For each insertion, the same frozen stoichiometry, the harmonized medium, the zero lower bound on growth and the parameters of the loopless formulation were used. The ordinary FBA maxima were reproduced before the additional checks. The recovery of flux feasibility was confirmed by a feasible flux distribution with an independent certificate for all active internal directions.

Sample	iCGB21FR	iCW773

Sample	iCGB21FR	iCW773
Full stoichiometry matched	538	640
Original EGC warning	13	23
Loopless flux in the EGC-free subset	406 / 525	343 / 617
Blocked in the EGC-free subset	119	274
EGC-positive with certified flux	13 / 13	23 / 23
Extended assessment, EGC flags retained	419 / 538 (77.9%)	366 / 640 (57.2%)

In iCW773, 21 positive results of ordinary FBA were blocked in addition: 9 parallel single-step, 1 path-shortening and 11 bridging variants. All these blocking events persisted when the technical bound $|G|$ was increased from 1000 to 10^6. In the conservative EGC-free subset of iCW773, 53 of 195 parallel single-step, 7 of 44 path-shortening and 214 of 378 bridging variants are blocked; $\chi^2(2) = 60.73$; $p = 6.49 \times 10^{-14}$. The complete row-by-row assessment is given in Table S33.

15.1. Existence of Flux and Its Global Maximum

For all 36 EGC-positive insertions, the existence of a positive solution without internal cycles was confirmed. Their global maxima were not proven within the time allotted: the optimization terminated at the time limit. Table S25 therefore contains the certified feasible flux as a lower bound, a separate flag `maximum_proven=false` and an empty field for the global maximum. Such a result makes it possible to classify an insertion as able to carry flux, but it does not allow the value found to be declared its limiting rate.

In the EGC control, 108 maximizations of ATP, NADH and NADPH consumption were performed after the bounds had been closed: 106 terminated optimally with an upper bound below 10^{-6}, and two stopped at the time limit. The largest optimal numerical value is 8.14×10^{-7}, below the accepted threshold. The separate 36 maximizations of the flux of the insertion itself in the closed system likewise did not yield a proven optimum within the time set. Zero intermediate solutions were not used as proof of a zero maximum. The conclusion that operation without cycles is possible is based on the positive open solutions and the independent certificates; the original EGC flags were retained.

Certified loopless feasibility is not a complete thermodynamic assessment. In particular, a mathematically feasible flux in the direction of the synthesis of a high-energy product does not confirm that this synthesis is physiologically feasible in the absence of data on ΔG and on concentrations. The extended assessment including the EGC variants is therefore reported separately from the conservative one and does not withdraw the warning about the direction or the energetics of the reaction.

15.2. Agreement Between the Models and the ArcE Control

Among the 355 shared fully matched variants without an original EGC warning, the loopless status agreed for 263 (74.1%). When all shared fully matched variants were included, the denominator increased to 378: 282 statuses agreed (74.6%), of which 245 permitted flux and 37 were blocked in both models. A further 85 permitted flux only in iCGB21FR and 11 only in iCW773. A separate Boolean flag in Table S34 marks the conservative 355-variant subset.

For the four control and ArcE-modified models at a growth rate of ≥0.05 h^{-1}, the removal of the cyclic components preserved all the previous L-arginine maxima, and the complete active sets passed the independent check of the directions. In iCGB21FR with Ddh added in the same way, the values remained 5.720543 and 7.019003 (+22.7%); in iCW773 with native DAPDH they remained 7.731907 and 8.696988 (+12.5%). This confirms that these product effects are not due to internal cyclic flux (Table S35).

16. Causes of the 17 Blocking Events in iCGB21FR

A total of 170 diagnostic interventions were tested: artificial sources and sinks for individual participants, the opening of existing exchanges, changes in the directions of neighboring reactions and targeted checks of MMM2. A positive result required a certified insertion flux of 10^{-4} mmol gDW^{-1} h^{-1}. A negative diagnostic result means the absence of a solution at this level and not an automatically proven zero maximum. The principal blocking of all 17 under the original conditions was proven separately in Section 14. The changes served to identify the constraints and were not introduced into the frozen models.

Rhea variants	Confirmed diagnostic recovery
22069, 23657, 15683, 67278, 30873, 32288	A joint independent source of the substrate and sink of the product of the main pair.
81193, 77737, 50706	Removal of aminoacetaldehyde, ITP or 1-propanol, respectively.
10982, 10294	Opening of EX_ser__D_e; for 10294, EX_acgam_e or EX_acmana_e.
50705, 13743	An independent source of 1-propanol or of indolepyruvate, respectively.
29045	A joint dTMP source and dUMP sink. Ports for the main pair THF/mlTHF alone gave no result.
57494	A sink for the co-product propionyl-CoA, or reversibility of MMM2.
27668, 61342	A sink for the corresponding methylmalonyl-CoA, or reversibility of MMM2.

The distribution of the closed-loop fluxes found among these 17 cases is 10 with two reactions, 6 with three reactions and 1 with four reactions; the cycles are therefore not all two-reaction cycles. The overall results of 117/17 are unchanged. Eight variants are the reverse directions of single native reactions with one-sided bounds; seven close a return path consisting of several reactions. For two variants, RHEA:50705 and RHEA:50706, the same full stoichiometry is already allowed in ALCD3/ALCD3ir. These two cases are marked as native equivalents in Tables S12 and S38: the absence of a Rhea annotation must not be equated with the absence of the chemical conversion.

16.1. What Can Be Regarded as a Limitation of the Reconstruction

Changing the bound of MMM2 alone restored flux for RHEA:27668, RHEA:61342 and RHEA:57494. For *C. glutamicum*, the B12-dependent conversion of methylmalonyl-CoA into succinyl-CoA has been demonstrated experimentally (Botella et al., 2009; https://doi.org/10.1128/JB.01756-08). This is a concrete reason for curating the direction of

MMM2 with regard to the availability of adenosylcobalamin; it does not mean that any diagnostic inversion of an irreversible reaction should be accepted as a correction.

For RHEA:10294 it is sufficient to open the modeled import of GlcNAc; however, no functional uptake of GlcNAc was observed in the wild type of *C. glutamicum* studied, and it was obtained by heterologous expression of *nagE* from *C. glycinophilum* (Matano et al., 2014; https://doi.org/10.1007/s00253-014-5676-9). Model-based recovery on this substrate therefore requires biological verification of the transport. The artificial ports for the other variants likewise point to a missing independent input or output of the network, but they do not prove that the corresponding reaction or transport process was omitted in error.

Of the 17 variants, nine can be assessed in a targeted manner in iCW773: seven remain blocked, whereas RHEA:77737 and RHEA:61342 permit flux with the EGC warning retained. For eight, an unambiguous mapping is lacking for at least one participant of the full stoichiometry. RHEA:22069 is not part of the main catalogue of iCW773 because of a native Rhea annotation, but a separate insertion made for diagnostic comparison confirmed the blocking. Such additional tests are not included in the denominator of 640 of the main catalogue (Table S36).

17. Main-Pair Assignment and Native Equivalence

Main pairs are assigned from the frozen Rhea equations after the compounds of the auxiliary-compound list are set aside. The list includes both iron ions, Fe^{2+} and Fe^{3+}: as a redox couple of identical elemental composition they would otherwise reach the maximum conserved-atom ratio and displace the metabolite conversion. Where a compound of the auxiliary list is itself the donor or acceptor of the transferred fragment, or where conserved-atom ranking selects a crossed pair or a minor fragment, the main pair is assigned by curation from the reaction chemistry. Table S9 lists the 32 directed variants with curated pairs, the basis of each assignment and the resulting status: six are in the two-anchor catalogue as curated main-pair overrides, twelve are one-anchor contexts, ten are excluded because the curated pair contains an auxiliary compound, two have no valid main pair and two have no context for their single matched metabolite. The two-anchor catalogue contains 846 variants and the one-anchor catalogue 3642.

Every fully mapped insertion equation was compared with every native model equation up to a non-zero stoichiometric scale factor. Equivalent native equations were found for 30 variants in iCGB21FR and 44 in iCW773; the corresponding direction is already permitted for 17 and 29, respectively. Table S11 records the proportionality coefficient and native bounds. These records expose incomplete Rhea annotation and curated nativity decisions; catalogue inclusion alone is not evidence of biological absence. They are retained explicitly within the annotation-based candidate catalogue.

The canonical release contains 92 bridging contexts with a PAPS/PAP main pair. All 92 are unassessed for flux because full stoichiometric mapping is unavailable. Adding both carriers to the auxiliary list as a separate projection sensitivity yields 427 graph reactions, 750 two-anchor and 3648 one-anchor contexts, with 268 parallel, 124 path-shortening and 358 bridging variants. Of the 92 canonical carrier-pair contexts, 90 are excluded and two become one-anchor contexts. This is a sensitivity of the graph representation and is not substituted for the canonical release. Table S10 records each disposition.

18. Robustness Across Media

Ordinary FBA was repeated for nine conditions: glucose baseline, fructose, gluconate, acetate, sucrose, oxygen-limited glucose, anaerobic glucose, ammonium-limited glucose and carbon-limited glucose. Carbon uptake limits are molar substrate rates, not carbon-equivalent rates. The baseline carbon and oxygen limits are each 10 mmol gDW^{-1} h^{-1}; oxygen limitation uses 2 and oxygen deprivation 0. Ammonium limitation uses 1 rather than 100; carbon limitation uses 2 rather than 10. Trace biotin remains at 0.001. No positive lower growth bound was imposed in this census.

All conditions have 538 fully mapped variants, 13 retained EGC exclusions and 525 conservative assessments. Eight conditions give 423 carrying and 102 blocked variants; sucrose gives 425 carrying and 100 blocked. The condition-dependent variants are RHEA:55082 and RHEA:70277. Across conditions, 423 variants always carry flux, 100 never do and two depend on sucrose supply. The class association remains significant in every condition. Loopless validation applies to the glucose baseline; alternative-medium verdicts are ordinary-FBA results. Tables S29–S30 provide all records.

19. ArcE Stoichiometry and Thermodynamic Sensitivity

ArcE: L-citrulline + NH_4^+ + ATP → L-arginine + ADP + phosphate + H^+. Native ArgG + ArgH: L-citrulline + L-aspartate + ATP → L-arginine + fumarate + AMP + pyrophosphate + H^+. ArcE uses ammonium and ATP-to-ADP conversion; the native route uses aspartate and ATP-to-AMP conversion. With pyrophosphate hydrolysis included, the latter consumes two ATP equivalents. Neither net equation directly consumes NADH or NADPH. The two reconstructions predict different absolute gains, 1.29846 and 0.96508 mmol gDW^{-1} h^{-1}, and different relative gains, 22.7% and 12.5%. These differences cannot be attributed solely to the baseline denominator.

Component contribution was calculated with equilibrator-api 0.8.1, equilibrator-cache 0.8.1, component-contribution 0.8.0 and equilibrator-cache-data 0.1.0 at pH 7.5, I = 0.25 M, pMg = 3.0 and 298.15 K (Beber et al., 2022, https://doi.org/10.1093/nar/gkab1106). The transformed equations omit buffered protons; water has unit activity. Concentration effects use $\Delta G' = \Delta G'^{\circ} + RT \ln Q$, $R = 8.314462618 \times 10^{-3}$ kJ mol^{-1} K^{-1}, with activities approximated by concentrations relative to 1 M.

Sensitivity inputs in mM are ATP 1–10, ADP 0.1–1, phosphate 1–20, pyrophosphate 0.01–0.5, AMP 0.01–0.5, ammonium 0.1–10, citrulline 0.01–1, arginine 0.1–10, aspartate 0.1–10 and fumarate 0.01–1. Endpoint combinations are enumerated independently for the six ArcE and seven native-net participants. ArcE has $\Delta G'^{\circ}$ = +7.59 ± 3.33, range −15.24 to +37.85 kJ mol^{-1}, with 10 of 64 endpoints negative. Native ArgG + ArgH without pyrophosphatase has $\Delta G'^{\circ}$ = +7.25 ± 3.28, range −61.25 to +9.52, with 126 of 128 endpoints negative. These fractions describe the chosen endpoint design, not a physiological probability. Adding pyrophosphatase gives a directly estimated $\Delta G'^{\circ}$ of −9.21 ± 3.28; at 1 mM for all non-water participants its mass-action value is −43.46 kJ mol^{-1}. No endpoint fraction is reported for this combined route.

Table S32 is a separate ATP/ADP-ratio sensitivity. ATP is fixed at 5 mM, ADP equals 5 divided by the ratio, citrulline is 0.1 mM, arginine 1 mM and phosphate 4.472 mM; ammonium is varied. Thus ADP spans 0.05–5 mM in this table. At ATP/ADP = 100 and ammonium 10 mM, $\Delta G'$ is −0.11; at ratio 10 and ammonium 1 mM it is +11.31 kJ mol^{-1}. Directionality depends on

all concentration terms in Q. These assumed concentrations are not measurements in *C. glutamicum*. The FBA optimization did not impose Gibbs-energy constraints.

The supplementary workbook contains 48 sheets, S0–S47. S9 covers the curated main-pair assignments, S10 the PAPS/PAP main-pair sensitivity, S11 complete native-equivalence matches, S29–S30 medium sensitivity, S31 thermodynamic reaction estimates and S32 the ratio sensitivity.
